# C3G Is Upregulated in Hepatocarcinoma, Contributing to Tumor Growth and Progression and to HGF/MET Pathway Activation

**DOI:** 10.3390/cancers12082282

**Published:** 2020-08-14

**Authors:** Celia Sequera, Paloma Bragado, Sara Manzano, Maria Arechederra, Sylvie Richelme, Alvaro Gutiérrez-Uzquiza, Aránzazu Sánchez, Flavio Maina, Carmen Guerrero, Almudena Porras

**Affiliations:** 1 Departamento de Bioquímica y Biología Molecular, Facultad de Farmacia, Universidad Complutense de Madrid, 28040 Madrid, Spain; celiasequera@ucm.es (C.S.); pbragado@ucm.es (P.B.); samanzan@ucm.es (S.M.); macalderon@unav.es (M.A.); alguuz@ucm.es (A.G.-U.); munozas@ucm.es (A.S.); 2 Instituto de Investigación Sanitaria del Hospital Clínico San Carlos (IdISSC), 28040 Madrid, Spain; 3 Departmento de Hepatología, Centro de Investigación Médica Aplicada (CIMA), Fundación para la Investigación Médica Aplicada, Universidad de Navarra, 31008 Pamplona, Spain; 4 Aix-Marseille Univ, CNRS, Developmental Biology Institute of Marseille (IBDM), Turing Center for Living Systems, UMR7288, Parc Scientifique de Luminy, 13009 Marseille, France; Sylvie.RICHELME@univ-amu.fr (S.R.); flavio.maina@univ-amu.fr (F.M.); 5 Instituto de Biología Molecular y Celular del Cáncer (IMBCC), Universidad de Salamanca-CSIC, 37007 Salamanca, Spain; 6 Instituto de Investigación Biomédica de Salamanca (IBSAL), 37007 Salamanca, Spain; 7 Departamento de Medicina, Universidad de Salamanca, 37007 Salamanca, Spain

**Keywords:** C3G, hepatocarcinoma, cancer, MET, cell signaling

## Abstract

The complexity of hepatocellular carcinoma (HCC) challenges the identification of disease-relevant signals. C3G, a guanine nucleotide exchange factor for Rap and other Ras proteins, plays a dual role in cancer acting as either a tumor suppressor or promoter depending on tumor type and stage. The potential relevance of C3G upregulation in HCC patients suggested by database analysis remains unknown. We have explored C3G function in HCC and the underlying mechanisms using public patient data and in vitro and in vivo human and mouse HCC models. We found that C3G is highly expressed in progenitor cells and neonatal hepatocytes, whilst being down-regulated in adult hepatocytes and re-expressed in human HCC patients, mouse HCC models and HCC cell lines. Moreover, high *C3G* mRNA levels correlate with tumor progression and a lower patient survival rate. C3G expression appears to be tightly modulated within the HCC program, influencing distinct cell biological properties. Hence, high C3G expression levels are necessary for cell tumorigenic properties, as illustrated by reduced colony formation in anchorage-dependent and -independent growth assays induced by permanent C3G silencing using shRNAs. Additionally, we demonstrate that C3G down-regulation interferes with primary HCC tumor formation in xenograft assays, increasing apoptosis and decreasing proliferation. In vitro assays also revealed that C3G down-regulation enhances the pro-migratory, invasive and metastatic properties of HCC cells through an epithelial-mesenchymal switch that favors the acquisition of a more mesenchymal phenotype. Consistently, a low C3G expression in HCC cells correlates with lung metastasis formation in mice. However, the subsequent restoration of C3G levels is associated with metastatic growth. Mechanistically, C3G down-regulation severely impairs HGF/MET signaling activation in HCC cells. Collectively, our results indicate that C3G is a key player in HCC. C3G promotes tumor growth and progression, and the modulation of its levels is essential to ensure distinct biological features of HCC cells throughout the oncogenic program. Furthermore, C3G requirement for HGF/MET signaling full activation provides mechanistic data on how it works, pointing out the relevance of assessing whether high C3G levels could identify HCC responders to MET inhibitors.

## 1. Introduction

Hepatocellular carcinoma (HCC) is the sixth most common cancer worldwide and the second deadliest cancer for men and sixth for women [1]. HCC results as a neoplastic transformation of hepatocytes and/or progenitor cells [2] that can go through an epithelial to mesenchymal transition (EMT) acquiring pro-migratory properties [3]. HCC heterogeneity and its late diagnosis are relevant factors for the lack of effective therapies and recurrence.

The receptor tyrosine kinase MET is frequently overexpressed in HCC [4], activated in about 50% of HCC patients, and linked to an aggressive phenotype [5,6]. Accordingly, a recent report based on a meta-analysis of tumors from patients suffering a surgical resection showed a significant poor prognosis associated to MET overexpression, due to a higher recurrence [7]. The implication of c-MET in HCC has prompted a number of clinical trials using MET targeting agents (alone or in combination) for HCC treatment in recent years [8,9,10].

C3G (Crk SH3-domain-binding guanine-nucleotide-releasing factor) (*RapGEF1*) is a guanine nucleotide exchange factor (GEF) for Rap and other Ras proteins [11,12,13]. However, some of its functions are independent of its GEF activity and rather rely on protein–protein interactions and/or its ability to translocate to the nucleus [14,15,16,17,18]. C3G is essential during embryogenesis for its role in cell adhesion [13]. In addition, it regulates other cellular functions, including migration, apoptosis and differentiation [19,20,21,22,23]. C3G plays also a relevant role controlling cell–cell interactions [24,25], through its binding to intracellular E-cadherin, leading to E-cadherin recruitment to the plasma membrane [24].

C3G function in cancer is controversial and appears to be dependent on the cellular context, tumor type and stage. In mouse fibroblasts, C3G prevents malignant transformation induced by several oncogenes [14,26]. Accordingly, C3G expression is reduced in cervical squamous cell carcinoma [27]. Nevertheless, C3G plays a dual role in colon carcinoma (CRC). C3G inhibits migration and invasion by down-regulating p38α activity, whereas it promotes tumor growth through p38α independent mechanisms [28]. In highly invasive breast carcinoma cells, C3G reduces migration [29]. In agreement with its potential role as an oncogene, C3G levels increased in human non-small-cell lung cancer [30]. Moreover, over-expression of p87C3G isoform is associated with chronic myeloid leukemia development [31]. Our previous genomic and transcriptomic studies using available human cancer databases indicated that *C3G* mRNA levels are increased in HCC compared to a normal liver [32]. Furthermore, HCC patients bearing somatic mutations and other genetic alterations in *C3G* gene showed lower survival [32]. Although these data suggest an implication of C3G in HCC, it remains unknown whether C3G is a positive or negative regulator of HCC cellular properties. Additionally, it remains unknown how C3G influences signaling in HCC.

Here, we employed in vitro and in vivo approaches to explore the role of C3G in HCC. We used human HCC cell lines and mouse HCC cell lines derived from the *Alb-R26^Met^* mouse HCC model, proven to be clinically relevant [33,34,35,36,37]. In addition, we have analyzed data from human HCC patient samples available in public databases to strengthen the potential relevance of C3G in HCC.

## 2. Results

### 2.1. C3G Is Overexpressed in Mouse and Human HCC

**Figure 1:**
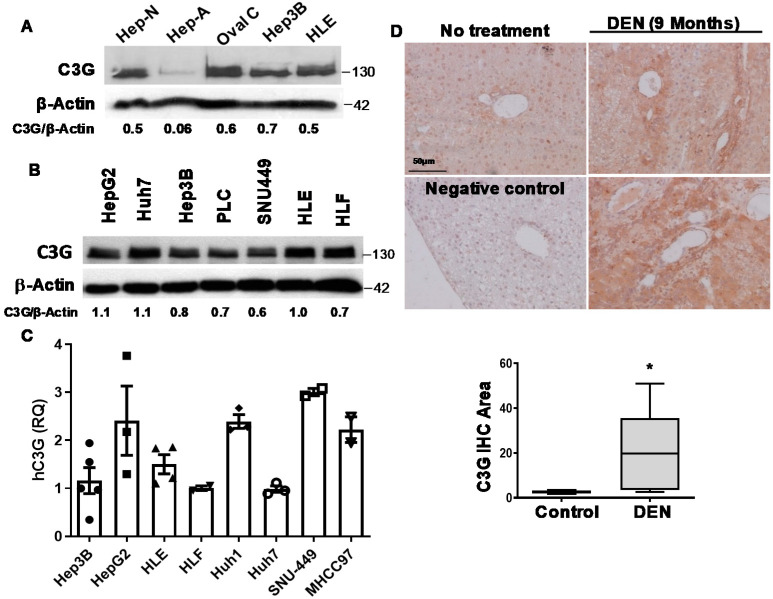

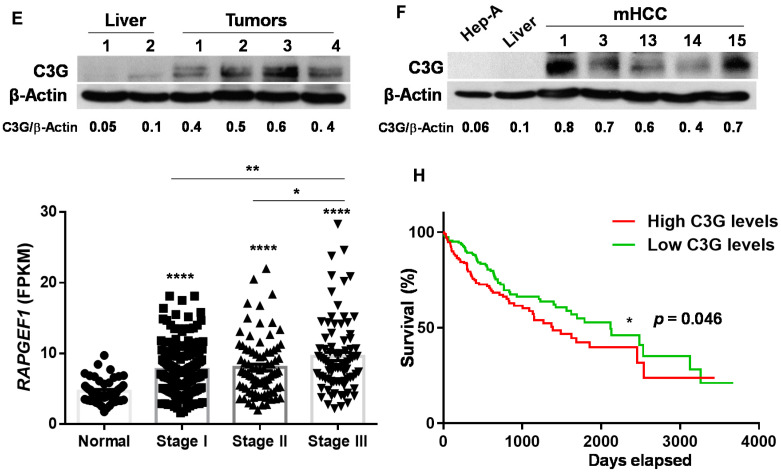
C3G expression is increased in HCC. (**A** and **B**) Western-blot analysis of C3G levels normalized with β-actin: (**A**) in neonatal hepatocytes (Hep-N), adult hepatocytes (Hep-A), oval cells (Oval C) and HCC cell lines (Hep3B and HLE) (*n* = 4); and (**B**) in a panel of human HCC cell lines (*n* = 2). (**C**) *C3G* mRNA levels in human HCC cell lines determined by RT-qPCR. RQ values are relative to Hep3B cells (*n* = 3). (**D**) Upper panels: representative images of C3G levels analyzed by immunochemistry in liver sections from control (no treatment) and DEN-treated mice at 9 months after treatment; lower panel: box plot showing the mean ± S.E.M. of C3G positive areas, analyzed by ImageJ. Negative control was incubated only with a secondary antibody. * *p* ≤ 0.05, using Student’s *t*-test, control vs. DEN-treated (*n* = 4–7). (**E**,**F**) Western-blot analysis of C3G protein levels normalized with β-actin in: (**E**) control healthy liver and *Alb-R26^Met^* tumors (1, 2, 3, 4); (**F**) adult hepatocytes (Hep-A), control healthy liver, and HCC cells established from *Alb-R26^Met^* HCC tumors (mHCC1, 3, 13, 14, and 15) (*n* = 3). (**G**) Scatter plot with bars shows mean ± S.E.M. of *C3G* (*RapGEF1)* mRNA levels in FPKM units (fragments per kilobase of exon model per million reads mapped) in normal healthy liver samples (50) and in HCC corresponding to different stages of disease progression: stage I (176 patients), stage II (84 patients), and stage III (83 patients). * *p* ≤ 0.05, ** *p* ≤ 0.01, **** *p* ≤ 0.0001, analyzed by Kruskal–Wallis followed by FDR, vs. normal samples or as indicated. (**H**) Kaplan–Meier survival curve shows patient survival (as percentage) vs. the days elapsed of two groups of HCC patients, according to high or low *C3G* mRNA levels. * *p* ≤ 0.05, using Log-Rank Mantel–Cox survival analysis.

Our previous analysis using public databases revealed an increase in *C3G* mRNA levels in patient tumor liver samples as compared to non-pathological liver [32], which suggests that C3G might play a role in HCC. Hence, in this new study, we first assessed C3G protein expression in a panel of human HCC cell lines as compared to mouse hepatocytes and liver progenitor cells (oval cells). High C3G protein levels were found in mouse neonatal hepatocytes (Hep-N) and oval cells, while adult hepatocytes displayed almost undetectable levels (Hep-A; Figure 1A). Remarkably, high C3G protein levels were found in all human HCC cell lines (Figure 1A,B). Consistent with protein data, RT-qPCR analyses revealed high *C3G* mRNA levels in a representative panel of human HCC cell lines (Figure 1C). This is also supported by public databases, which show that human HCC cell lines and progenitor cells present higher *C3G* mRNA levels than adult hepatocytes (Figure S1A). Additionally, we detected high C3G protein levels in mouse Diethylnitrosamine (DEN)-induced liver tumors, both after 9 months (Figure 1D) and 12 months of DEN treatment (Figure S1B), when all the mice presented visible tumors. Moreover, the analysis performed using databases also revealed an increase in *C3G* mRNA levels in livers from DEN treated mice (Figure S1C). Next, we evaluated C3G expression levels in liver tumors and HCC cell lines (mHCCs) derived from the *Alb-R26^Met^* mouse HCC model induced by moderately increased MET levels in hepatocytes, which recapitulates the proliferative subtype of human HCC [33,34,35,36,37]. As shown in Figure 1E, C3G overexpression was found in all tumors as compared to normal liver tissue. Similarly, high C3G protein levels were observed in HCC cell lines (mHCCs) derived from *Alb-R26^Met^* liver tumors (Figure 1F), in parallel with increased Met and P-MET levels (Figure S1D).

Next, we used different public databases to explore *C3G* mRNA levels in human liver tumors from different progression stages compared to non-pathological liver samples. We found that *C3G* (*RapGEF1*) mRNA levels in patients from stages I to III were significantly increased as compared to normal liver (Figure 1G). This is in agreement with our previous studies [32] showing a higher *C3G* mRNA expression in HCC samples, where progression stages were not considered. Furthermore, in this new study, a significant increase in *C3G* mRNA levels was detected in HCC patients from stage III as compared to those from stages I and II (Figure 1G). Finally, a Kaplan–Meier survival curve showed that higher *C3G* mRNA levels correlated with worse prognosis (Figure 1H), when considering all the patients together. However, when each stage is analyzed separately, differences are only significant at stage III [38]. Together, these data indicate that C3G is highly expressed in liver progenitor cells and immature hepatocytes, then down-regulated in adult hepatocytes and re-expressed in HCC, being progressively up-regulated during tumor progression, reaching a very high level at advanced stages.

### 2.2. C3G down-Regulation Reduces Foci Formation by HCC Cells

**Figure 2:**
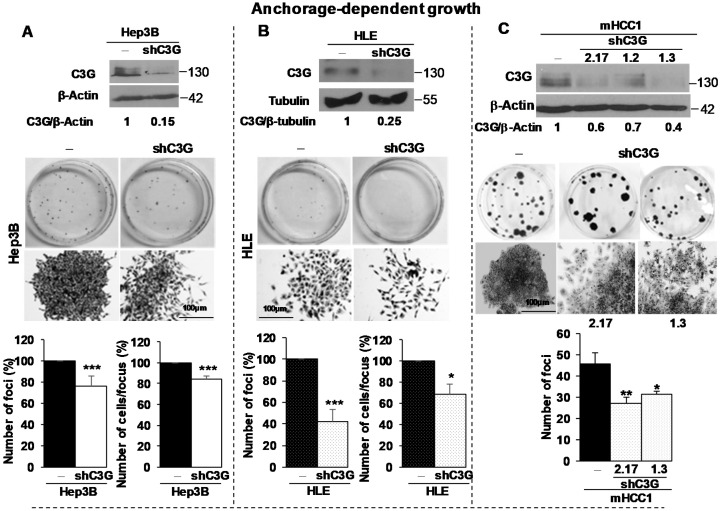

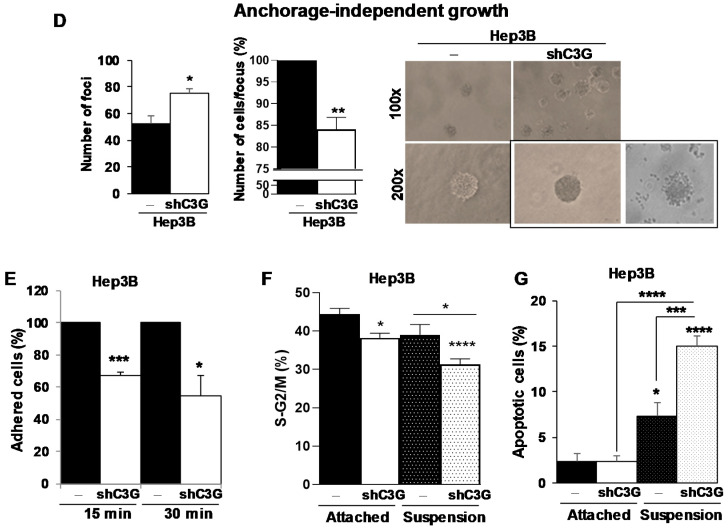
C3G knock-down reduces in vitro tumorigenic properties of HCC cells. Hep3B and HLE non-silenced (−) and C3G knock-down (shC3G) cells; and mHCC1 (non-silenced (−) and C3G knock-down (clones 2.17, 1.2 and 1.3)) were maintained in complete medium. (**A**,**B**) Top panels: Western-blot analysis of C3G expression in HCC cells, normalized with β-actin or tubulin. Middle panels: Representative macroscopic (whole dish) and microscopic (individual foci) photographs of anchorage-dependent growth assay. Bottom panels: Histograms show the mean ± S.E.M. of the number of foci (%) and the number of cells per focus (%). (**C**) Upper panels: Western-blot analysis of C3G expression in mHCC1 cells, normalized with β-actin. Middle panels: Representative macroscopic (whole dish) and microscopic (individual foci) photographs of anchorage-dependent growth assay. Lower panel: Histogram shows the mean ± S.E.M. of the number of foci. (**D**) Anchorage independent growth of Hep3B cells. Left panel: Histograms represent the mean ± S.E.M. of the number of foci (left) and cells per focus (right). Right panels: Representative microscope images of foci formation. (**E**) Adhesion assay in Hep3B cells. Histogram represents the mean ± S.E.M. of the percentage of adhered cells (%) after 15 and 30 min. Individual cells were counted with ImageJ. (**F**) Histograms showing the mean ± S.E.M. of the percentage of Hep3B cells in S+G2/M phases or (**G**) with a lower DNA content than 2C (apoptotic) analyzed by cytometry, when maintained in culture with 10% serum under adherent or suspension conditions for 6h. * *p* ≤ 0.05, ** *p* ≤ 0.01, *** *p* ≤ 0.001, **** *p* ≤ 0.0001, vs. non-silenced cells (−), (**A**,**B**,**D**,**E**) analyzed by Student’s *t*-test, (**A**,**B**) *n* > 3 or (**C**,**F**,**G**) one-way ANOVA; *n* ≥ 3.

We next explored whether C3G overexpression in HCC cells contributed to their tumorigenic properties through a permanent C3G silencing using various shRNAs targeting sequences. We used three human HCC cell lines with distinct genetic alterations and phenotypes: Hep3B and Huh7 epithelial-like and HLE mesenchymal-like cells. Moreover, we silenced C3G in *Alb-R26^Met^* HCC cells (mHCC1). C3G silencing in Hep3B, Huh7 and HLE cells led to a 50 to 85% C3G protein down-regulation (Figure 2A,B and Figure S2A). Similarly, shRNA silencing in mHCC1 cells reduced C3G levels to 30–60%, depending on clones (Figure 2C). C3G down-regulation reduced foci number in Hep3B, HLE, Huh7 (Figure 2A,B and Figure S2B) and mHCC1 cells (Figure 2C) in anchorage-dependent growth assays.

The foci formed by Hep3B, HLE and Huh7 cells with low C3G levels showed reduced cell density, a cell scattered morphology, and an apparent decrease in cell–cell interactions (Figure 2A,B and Figure S2B). This was also evident in anchorage-independent growth assays performed with Hep3B cells, in which a significant reduction in the number of cells per colony was accompanied by a subsequent increased number of colonies, likely due to cells moving away to generate new foci (Figure 2D).

To further understand how C3G overexpression influences cellular and biological properties of HCC cells, we performed complementary studies to evaluate adhesion, proliferation and apoptosis. We found that C3G down-regulation reduced adhesion in Hep3B cells (Figure 2E), in agreement with the presence of fewer foci with a lower number of cells in anchorage-dependent growth assays. Furthermore, we explored whether C3G down-regulation influenced the capability of cells to proliferate and survive under adherent and non-adherent conditions. We found a significant decrease in proliferation in C3G silenced Hep3B cells, both under adherent and non-adherent conditions (Figure 2F). Moreover, we observed an increase in the number of apoptotic cells when Hep3B^shC3G^ cells were maintained under suspension conditions, compared to non-silenced cells (Figure 2G and Figure S2C,D). In addition, under adherent conditions, active caspase 3 levels were also significantly increased and the number of apoptotic nuclei tended to be higher in Hep3B cells with C3G down-regulation (Figure S2C,D).

Collectively, these findings indicate that C3G is relevant for HCC cell adhesion and cell survival. In addition, C3G promotes cell proliferation. All this might contribute to promote in vitro clonal growth of HCC cells.

### 2.3. C3G Down-Regulation Enhances Migratory and Invasive Properties of HCC Cells by Switching from an Epithelial to a Mesenchymal Phenotype

**Figure 3:**
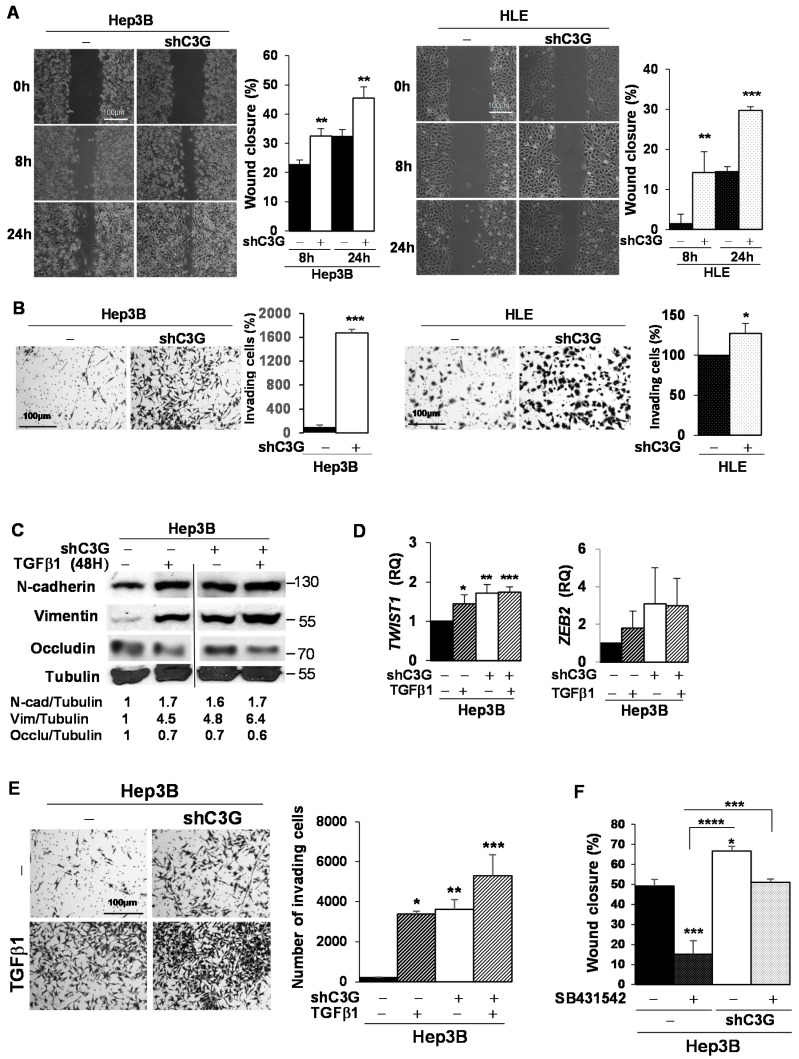
C3G knock-down increases Hep3B and HLE migratory and invasive properties. Hep3B and HLE non-silenced (−) and C3G knock-down (shC3G) cells were used. (**A**) Representative microscope images after 0, 8, and 24h of migrating cells in wound healing assays. Histograms show the mean ± S.E.M. of wound closure (%). (**B**) Invasion assay using 10% FBS as chemoattractant. Representative microscope images of invading cells. Histograms show the mean ± S.E.M. of invading cells (%). (**C**–**E**) Hep3B cells were maintained untreated (−) or were either treated with TGF-β1 for 48 h to induce EMT (**C**,**D**) or chronically to maintain mesenchymal phenotype (**E**). (**C**) Western-blot analysis of mesenchymal markers, N-cadherin and Vimentin, normalized with Tubulin, *n* = 3. (**D**) RT-qPCR analysis of *TWIST1* and *ZEB2* mRNA levels. Histograms show the mean ± S.E.M. of RQ values referred to the control (non-silenced and non-treated cells). (**E**) Invasion assay through matrigel using 10% FBS as chemoattractant. Left panels: representative microscope images of invading cells after 24 h. Right panel: histogram show the mean ± S.E.M. of the number of invading cells. (**F**) Histogram represents the mean ± S.E.M. of the percentage of wound closure after 6 h in the absence of serum, either with (+) or without (−) SB431542, a TGF-β1 inhibitor. * *p* ≤ 0.05, ** *p* ≤ 0.01, *** *p* ≤ 0.001, **** *p* ≤ 0.0001, vs. non-silenced cells (−) non-treated (−), (**A**,**B**) Student’s *t*-test, (**D**) One-way ANOVA and (**E**,**F**) Two-way ANOVA, both followed by multiple comparison; *n* ≥ 3.

Next, we explored whether the adhesion changes observed in HCC cells following C3G down-regulation would influence migration and invasion properties. By performing wound healing assays, we found that C3G down-regulation enhanced migration of Hep3B and HLE cells (Figure 3A). Moreover, matrigel invasion assays revealed an exacerbated invasive capacity of Hep3B^shC3G^ and HLE^shC3G^ cells compared to non-silenced cells (Figure 3B). This was particularly evident in Hep3B cells, characterized by a low invasive capability linked to their epithelial-like phenotype.

The drastic change in the migratory and invasive properties of HCC cells with down-regulated C3G expression prompted us to analyze whether these cells acquired mesenchymal markers, while they lost epithelial ones, under these conditions. We found that protein levels of the mesenchymal markers, Vimentin and N-Cadherin, were up-regulated in Hep3B cells (epithelial-like) upon C3G down-regulation (Figure 3C), reaching levels comparable to those found in non-silenced cells upon a short-term treatment with TGF-β1, a well-known inducer of EMT in HCC cells [39,40,41]. In addition, Occludin, a tight junction protein, was also down-regulated upon both C3G silencing and TGF-β1 treatment (Figure 3C). Moreover, β-Catenin, a known EMT inducer, which was localized in the membrane of non-silenced Hep3B cells, was internalized upon C3G silencing (Figure S3A). In HLE cells (mesenchymal-like), C3G down-regulation was accompanied by increased Vimentin levels, reaching similar levels to those induced by TGF-β1 (Figure S3B). Next, mRNA levels of *TWIST1* and *ZEB2*, two EMT-inducing transcription factors, were quantified. *TWIST1* mRNA levels significantly increased in Hep3B^shC3G^ cells to a similar extend to that induced by TGF-β1 in non-silenced Hep3B cells (Figure 3D). TGF-β1 did not further enhance *TWIST1* expression in Hep3B^shC3G^ cells. *ZEB2* mRNA levels showed a similar tendency.

To further assess whether C3G down-regulation promotes the acquisition of a mesenchymal phenotype responsible for the enhanced HCC cell migration and invasion, we studied the effect of a long-term treatment with TGF-β1, known to lead to a stable EMT in these cells [41]. Long-term treatment with TGF-β1 highly increased the invasion of non-silenced Hep3B up to the level found in untreated C3G silenced Hep3B cells (Figure 3E). Moreover, enhanced invasion of Hep3B^shC3G^ cells was further increased by TGF-β1 (Figure 3E).

Overall, these results indicate that C3G down-regulation in HCC cells led to changes in the expression of EMT-associated genes and the acquisition of a pro-migratory and invasive phenotype reminiscent of the TGF-β-induced EMT. However, the fact that TGF-β1 has an additional effect over that elicited by C3G silencing increasing Vimentin expression and cell invasion, suggest that C3G down-regulation could be acting both through TGF-β dependent and independent mechanisms. Hence, we analyzed the effect of a TGF-β receptor inhibitor (SB431542) on cell migration. Treatment with this inhibitor significantly decreased migration in non-silenced cells, but not in Hep3B^shC3G^ cells (Figure 3F). This suggests that C3G down-regulation promotes an EMT-like process through a mechanism that might be, at least, partially independent of TGF-β signaling.

### 2.4. C3G down-Regulation Reduces Primary HCC Tumor Growth

**Figure 4:**
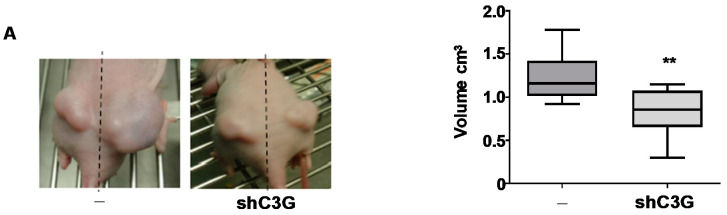

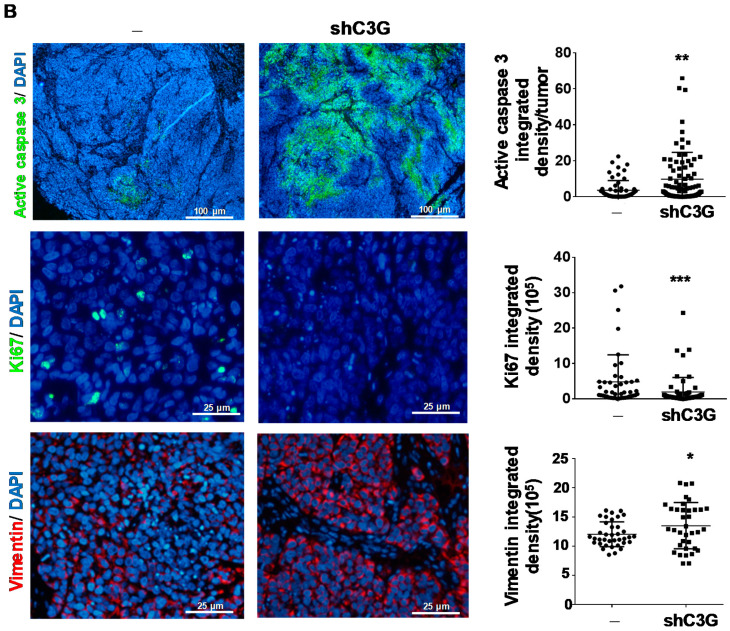
C3G down-regulation interferes with in vivo tumor growth. Xenograft assay was performed by subcutaneously injection of Hep3B (non-silenced (−) and C3G knock-down (shC3G)) cells into nude mice flanks. (**A**) Left panel: representative images of mice flanks showing tumors at the end point. Right panel: box plot shows the mean ± S.E.M. of tumor volume in cm^3^ at the end point (3 weeks). (**B**) Left panels: representative microscope images of immunofluorescence analysis of active caspase 3 (green), ki67 (green), Vimentin (red) and nuclei DAPI staining (blue) in paraffin-embedded tumor sections. Right panels: scatter plots showing the mean ± S.E.M. of positive area/tumor for each marker analyzed as integrated density/DAPI area. * *p* ≤ 0.05, ** *p* ≤ 0.01, *** *p* ≤ 0.001, vs. non-silenced cells (−), using Student’s *t*-test (for A and for Vimentin in B,) or Mann–Whitney test (for active Caspase 3 and Ki67 in B); *n* = 2 (two independent experiments with 8 nude mice per experiment).

We next explored whether C3G down-regulation affected tumorigenic properties of HCC cells in vivo by performing xenografts assays. C3G silenced and non-silenced Hep3B cells were injected into nude mice flanks. We found that the volume of the tumors generated by Hep3B^shC3G^ cells was significantly reduced, compared to those originated by control cells (Figure 4A). Next, we evaluated apoptosis and proliferation in tumors. We found that active caspase 3-stained area was significantly larger in tumors generated by C3G silenced cells than in those originated by non-silenced cells, while the number of Ki67-positive cells was significantly reduced (Figure 4B). These results indicate that C3G down-regulation reduced in vivo tumor growth of HCC cells through a mechanism that involves both an up-regulation of apoptosis and a decrease in cell proliferation. Additionally, Vimentin levels were increased in tumors generated by Hep3B^shC3G^ cells (Figure 4B), coherent with its up-regulation in cultured cells (Figure 3C).

Based on the increased migration and invasion found in HCC cells with C3G down-regulation (Figure 3), accompanied by higher levels of mesenchymal markers such as Vimentin, we explored whether the dissemination properties of cells with reduced levels of C3G were increased. Hence, we analyzed the presence of disseminated tumor cells (DTCs) in the bone marrow of mice used for xenograft assays. We found that the number of DTCs tended to be higher in mice injected with C3G-silenced cells (Figure S4). This supports the existence of a potential increased metastatic capacity of cells expressing low C3G levels.

### 2.5. C3G Levels Inversely Correlate with Lung Metastasis Formation by Mouse Alb-R26^Met^ HCC Cells with Enhanced MET Expression

**Figure 5:**
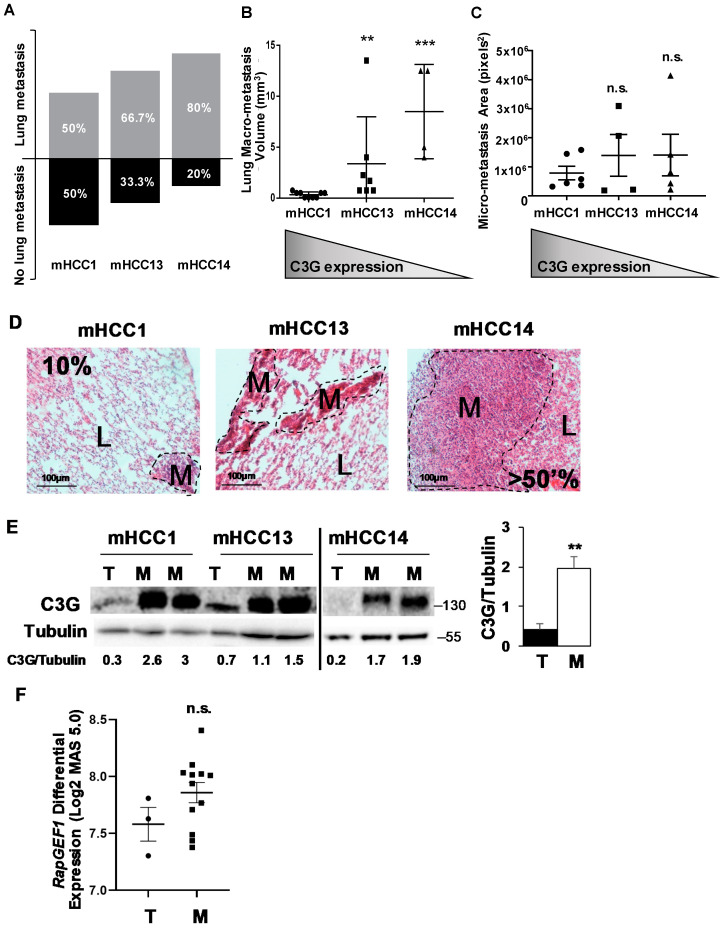
C3G expression inversely correlates with the lung metastatic potential of mouse *Alb-R26^Met^* HCC cell lines. Mouse Alb-R26Met HCC cell lines (mHCC1, mHCC13, and mHCC14) were injected subcutaneously into mice flanks to generate tumors and lung metastasis. (**A**) Histogram showing the percentage of mice with and without lung metastasis for each mHCC cell line. (**B**,**C**) Scatter plots show the mean ± S.E.M of the volume (mm^3^) of lung macro-metastases for each mHCC cell line (B) or the lung micrometastasis area (pixels^2^) for each mHCC cell line (**C**), analyzed in Haematoxilin/Eosin-stained paraffin-embedded lung sections using ImageJ. Kruskal–Wallis test was used. Grey triangle shows the corresponding decreasing levels of C3G in mHCC cell lines. (**D**) Representative images of the Hematoxylin/Eosin stained lung (L) and metastasis (M). Dotted lines delimit metastasis area. ** *p* ≤ 0.01, *** *p* ≤ 0.001 vs. mHCC1, using Kruskal–Wallis test followed by multiple comparison, *n* = 2. (**E**) Western-blot analysis of C3G levels in tumors and lung metastasis generated by mHCC cells normalized with Tubulin. Histogram represents the mean ± S.E.M of the quantification of C3G in several tumors and metastasis (relative values). ** *p* ≤ 0.01, tumors vs. metastasis using Student-t test, *n* = 4 (**F**) Scatter plot represent mean ± S.E.M. of *RapGEF1* mRNA differential expression levels in log2 MAS 5.0 signal of HCC primary tumors and lung metastasis from HCMDB (Human Cancer Metastasis Data Base) analyzed by Student’s *t*-test (*p* = 0.17) vs. primary tumor. n.s.: non-significant.

According to the in vitro data, C3G down-regulation increased pro-migratory and invasive properties of HCC cells. In addition, Hep3B^shC3G^ cells appeared to have an enhanced dissemination capability to bone marrow when subcutaneously injected in nude mice. Hence, we further explored in vivo whether C3G levels influence metastatic features of HCC cells using a panel of *Alb-R26^Met^* HCC cells with enhanced MET levels. We used mHCC1, mHCC13 and mHCC14 cells, covering a range from high to low C3G expression (Figure 1F). These cells were subcutaneously injected into mice flanks and once the primary tumors grew, they were surgically removed, and the mice were maintained to follow lung metastasis development. We found that the percentage of mice presenting lung metastasis was inversely correlated with C3G protein levels of *Alb-R26^Met^* HCC cells (Figure 1D and Figure 5A), being 50% for mHCC1, 66.7% for mHCC13, and 80% for mHCC14 cells. The volume of lung macro-metastases also showed an inverse correlation with C3G protein levels (Figure 5B). Micro-metastases areas were also higher in lung tumors originated from mHCC13 and mHCC14 cells (Figure 5C,D). Surprisingly, C3G protein levels were increased in all lung metastases compared to primary xenograft tumors (Figure 5E and Figure S5). These results suggest that, whereas low levels of C3G correlate with enhanced *Alb-R26^Met^* cell metastatic properties, C3G re-expression is associated with the growth of these secondary lung tumors. In agreement with this, using public databases, we found that *C3G* mRNA levels tend to be higher in lung metastasis samples (the most prevalent type of metastasis) from HCC patients (Figure 5F).

### 2.6. C3G Ensures Full Activation of the HGF/MET Signaling Pathway in Human HCC Cells

**Figure 6:**
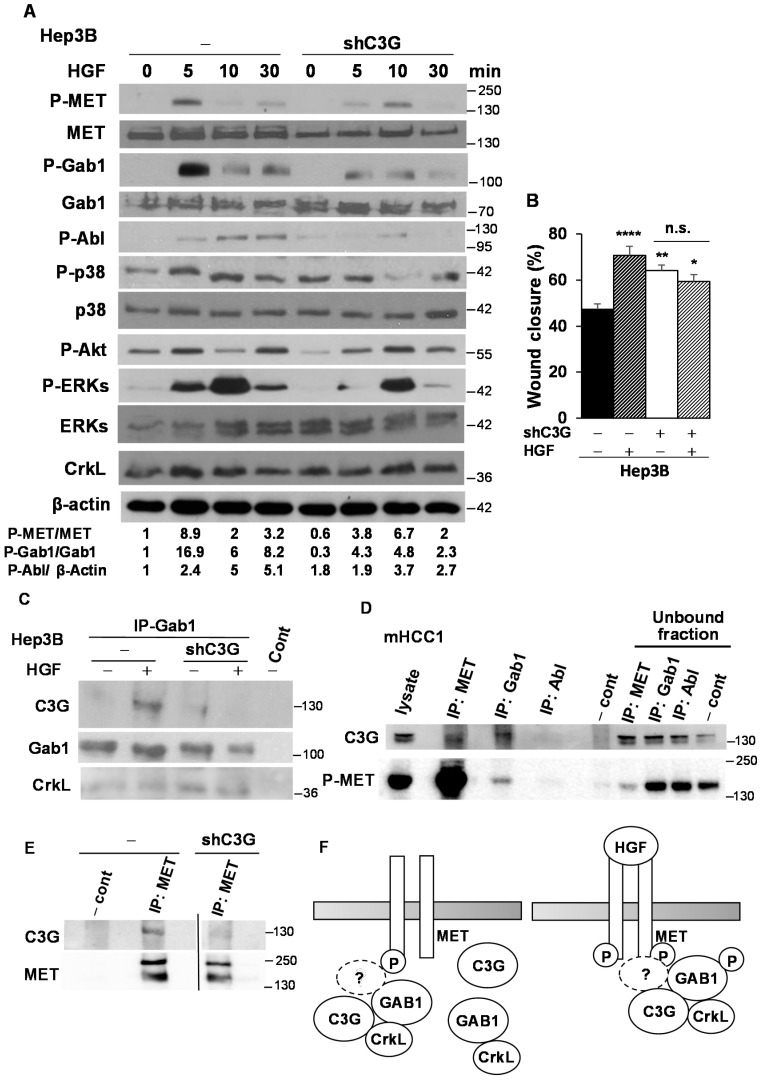
HGF/ MET signaling is defective in C3G knock-down HCC cells. Hep3B (non-silenced (−) and C3G knock-down (shC3G)) were serum starved for 24h and stimulated with HGF. (**A**) Analysis of the activation of MET and its downstream effectors after HGF stimulation at the indicated time points (min). Western-blot analysis of P-MET, MET, P-Gab1, Gab1, P-Abl, P-p38, p38, P-Akt, P-ERKs, ERKs, CrkL normalized with β-actin (P-Abl) or its corresponding total protein (P-MET and P-Gab1) levels, *n* = 3. (**B**) Wound healing assay. Histogram shows the mean ± S.E.M. of wound closure in cells maintained in the absence of serum, untreated or treated with HGF for 24 h. Two-way ANOVA followed by multiple comparison, * *p* ≤ 0.05, ** *p* ≤ 0.01, *** *p* ≤ 0.001, *n* ≥ 3. (**C**) Immunoprecipitation of Gab1 (IP-Gab1) in Hep3B lysates and western-blot analysis of C3G, Gab1 and CrkL in untreated or HGF (5min) treated cells (*n* = 4). (**D**) Immunoprecipitation of MET (IP: MET), Gab1 (IP: Gab1) and Abl (IP: Abl) from lysates of mouse *Alb-R26^Met^* mHCC1 cells, followed by western-blot analysis of C3G and P-MET (Y1234/Y1235) in IPs and in the unbound fraction (*n* = 2). (**E**) Immunoprecipitation of MET (IP: MET) in lysates from mHCC1 control (−) or shC3G silenced (shC3G) cells, followed by western-blot analysis of C3G and MET. As a negative control, sepharose beads were incubated with lysates in **C**–**E**. (**F**) Scheme showing the complexes formed in untreated (Left) or HGF treated cells (Right). MET can bind C3G when phosphorylated in a ligand independent manner.

We have shown that C3G down-regulation decreased anchorage-dependent growth of *Alb-R26^Met^* mHCC1 cells, which have increased MET levels and high C3G expression. In addition, previous published studies [42,43,44] proposed C3G and/or its main interactor, Crk, as major mediators of HGF/MET signaling in other cell types. We, therefore, explored whether C3G might play a role in HGF/MET signaling in HCC cells. We evaluated the impact of C3G silencing on MET signaling activation in response to HGF in Hep3B cells by analyzing the phosphorylation of MET and Gab1, an important adaptor for MET signaling. We found that MET and Gab1 phosphorylation was strongly reduced and delayed in Hep3B^shC3G^ cells (Figure 6A). Consistently, the HGF-induced phosphorylation of Abl, p38 MAPK, and ERKs also decreased in C3G-silenced cells (Figure 6A). Furthermore, we found that, in contrast to control cells, where HGF enhanced migration, Hep3B^shC3G^ cells failed to migrate in response to HGF stimulation (Figure 6B). Together, these results indicate that HGF/MET signaling is defective in C3G-silenced cells.

Crk is involved in mediating some HGF actions through its interaction with Gab1 [42,43,44]. Moreover, in HEK293 cells overexpressing Gab1, HGF induced the association of Gab1 to the preformed CrkL-C3G complex through CrkL SH2 domain [42]. Therefore, to understand the connection between C3G and HGF/MET signaling pathway, we explored the potential interaction between endogenous C3G and Gab1 in Hep3B cells and the effect of C3G silencing. We found that in response to HGF, C3G co-immunoprecipitated with Gab1 only in non-silenced cells (Figure 6C). However, CrkL-Gab1 association remained unaltered regardless of the presence of HGF, in both non-silenced and C3G-silenced cells (Figure 6C). This indicates that HGF induces the binding of Gab1 to C3G in Hep3B cells to mediate MET signaling, which supports the defective HGF/MET signaling found in C3G-silenced cells. However, CrkL would not be the necessary mediator, as it is bound to Gab1 in unstimulated cells with low levels of C3G. Strikingly, pull-down assays using GST bound to Crk SH3 domain revealed an interaction of Crk SH3 domain with P-Gab1 and P-MET in non-silenced Hep3B cells in response to HGF (Figure S6A), either directly through the Gab1 proline-rich domain or through an adaptor protein. On the other hand, treatment of Hep3B cells with HGF induced the binding of P-Gab1, but not CrkL, to the proline rich domain of C3G (C3GSH3b) in pull-down assays (Figure S6B). Hence, C3G might use an alternative adaptor with SH3 domains to mediate its binding to P-Gab1.

To further understand the mechanisms involved in the defective HGF/ MET signaling in C3G silenced HCC cells, we performed additional immunoprecipitations in mouse HCC cell lines with increased MET and P-MET levels (Figure S1D). As shown in Figure 6D, C3G was present in MET and Gab1 immunoprecipitates. Additionally, phosphorylated MET was detected in Gab1 immunoprecipitates, indicating that active MET forms complexes, directly or indirectly, with C3G and Gab1. Moreover, C3G-MET interaction was highly reduced in C3G knock-down cells, as expected (Figure 6E). All these data support an interaction between active MET, C3G and Gab1, which might facilitate full activation of MET and the downstream pathways. On the other hand, previous published data indicate that E-cadherin can interact with MET, amplifying HGF-MET signaling [45,46,47]. C3G is also known to bind E-cadherin through its E-cadherin binding domain [47]. Therefore, we analyzed this interaction and we found that only upon HGF stimulation C3G interacted with the E-cadherin domain known to bind C3G in pull-down assays (Figure S6C). This supports the idea that E-cadherin might contribute to connect MET and C3G in response to HGF.

## 3. Discussion

In this work, we uncover a previously unknown function of C3G in HCC development and progression. We found that C3G protein levels increase in human and mouse HCC cell lines and in two models of mouse HCC, induced by either DEN treatment or enhanced MET expression in hepatocytes. This is in agreement with the higher expression of *C3G* mRNA previously found in HCC patients [32]. Our new analyses support that *C3G* mRNA expression is high in HCC patients and reveal a gradual increase during disease progression, leading to a very high C3G expression in advanced stages, associated with reduced survival. This points out to *C3G* as a new key player for HCC progression. This is also supported by our previous analyses showing that mutations and other genetic alterations, such as amplifications or deletions, in *C3G* gene are also associated with decreased patient survival [32].

In vitro and in vivo data derived from clonogenic and xenografts assays, respectively, indicate that C3G down-regulation reduces tumor growth by decreasing proliferation and survival of HCC cells, in agreement with the pro-tumorigenic function of C3G in CRC cells [28] and in human non-small-cell lung cancer [30].

As mentioned in the introduction, MET is often overexpressed in HCC patients and associated with higher recurrence and poor prognosis [4,5,6,7]. Therefore, the defective HGF/MET signaling observed upon C3G down-regulation might, at least, partially explain the reduced proliferation and survival of cells from HCC tumors bearing low levels of C3G, taking into account that Hep3B and HLE cells overexpress MET (Figure S7). Moreover, C3G silencing in mouse HCC cells with enhanced MET expression also decreases anchorage-dependent growth, which further supports the relevance of C3G as a potential mediator of MET-induced tumorigenic capacity.

Our data derived from the in vitro analyses, as well as the in vivo quantification of disseminated HCC cells to the bone marrow, indicate that low levels of C3G enhance migration and invasion, as previously described for CRC [28]. This might be a consequence of a switch from an epithelial to a mesenchymal phenotype, similar to the EMT induced by TGF-β [41] (Figure 3C–E), used as a positive control. However, the mechanisms used by C3G to regulate this process would be, at least, partially independent of TGF-β, as inhibition of its receptor did not significantly affect the migration of C3G-silenced HCC cells. p38 MAPK activation could also play a role as it is highly activated in C3G-silenced HCC cells (Figure S8A) and its inhibition, using a p38α/β inhibitor, decreases migration (Figure S8B). This is in agreement with the pro-invasive function of p38α MAPK in CRC [28] and HCC [48,49,50]. On the other hand, different from MCF-7 breast cancer cells, where β-catenin down-regulation mediates the inhibition of migration by C3G [51], β-catenin levels remained unchanged in C3G-silenced HCC cells [38]. However, the observed β-catenin internalization might favor an EMT-like process.

The pro-migratory effect of C3G down-regulation is in agreement with the higher frequency of lung metastasis observed in mice injected with mouse HCC cell lines presenting the lowest levels of C3G (mHCC14). Moreover, secondary lung tumors generated by these cells were larger. Concerning these data, it is important to mention that MET levels and the pro-tumorigenic potential were similar in mHCC13 and mHCC14 cell lines (Figure S1D) [34,35], suggesting that the different metastatic capacity would not be due to differences in MET expression. Surprisingly, although these highly metastatic HCC cell lines presented relatively low levels of C3G, a very high C3G expression was detected in all analyzed lung metastases (Figure 5E and Figure S5). This supports the idea that re-expression of high C3G levels might favor the growth of secondary tumors, at least, in the lung niche. This would be in agreement with data from patients who developed lung metastasis, which show a tendency of higher *C3G* mRNA levels than in primary tumors. This might explain why patients with high C3G expression have a lower survival. Future studies will be necessary to determine whether C3G is required for the growth of lung metastases.

Although some C3G actions are not dependent on its GEF activity, C3G performs important functions through its exchange activity on Rap. Thus, Rap could mediate C3G effects in HCC. However, there is no much information about Rap function in HCC, and that available is quite controversial [32]. Rap1 could suppress tumorigenesis in Hep3B cells [52] or contribute to HCC induction [53]. More recent studies reported an upregulation of either Rap2B or Rap1B [54,55] expression in HCC, associated with increased proliferation and migration. Although we have not determined whether Rap mediates C3G actions in the HCC cell lines used for our studies, Rap1 protein levels were rather low in Hep3B cells [38]. Moreover, based on published data, Rap1 plays an opposite function in Hep3B cells [52] to that uncovered here for C3G. Therefore, we would not expect Rap1 to be a major mediator of C3G, at least, in Hep3B cells.

Our data support an interaction between active MET, C3G and Gab1, necessary for full activation of MET and the downstream pathways (Figure 6F). Abl might also facilitate MET activation when C3G is present. Hence, although we did not detect Abl in MET or Gab1 immunoprecipitates, where C3G was present, C3G is known to interact with Abl [19,56]. Therefore, Abl might associate with MET through C3G, facilitating MET activation. According to this idea, HGF-induced MET phosphorylation decreases upon Abl inhibition with imatinib [38], in agreement with previous studies showing reduced MET phosphorylation in Abl-silenced cells [57]. On the other hand, previously published data indicate that in HEK293 cells overexpressing Gab1, following its phosphorylation in response to HGF, CrkL-C3G complex binds to P-Gab1, leading to Rap1 activation [42]. Instead, in Hep3B cells, we found an interaction between Gab1 and CrkL in untreated cells (Figure 6). This agrees with previous studies, where Crk was found to interact with Gab1 in unstimulated cells through its N-terminal SH3 domain [44]. Furthermore, C3G proline-rich domain associates with P-Gab1 in Hep3B cells treated with HGF, but not with CrkL (Figure S4A). Therefore, CrkL would not be the necessary mediator for C3G binding to Gab1. In fact, CrkL might be bound to Gab1 in unstimulated cells and C3G would interact with Gab1 only in response to HGF or when MET is constitutively phosphorylated, most probably through alternative mediators containing SH3 domains. Therefore, adaptors such as Grb2 or p130Cas might mediate the interaction between P-Gab1 and the C3G proline rich domain [43]. It is also conceivable that other proteins can also contribute to mediating C3G-P-Gab1 association; such as Abl, through its SH3 domain [19]. E-cadherin is another potential candidate, as both C3G and MET can interact with it [20,45,58]. For example, in MCF-7 breast cancer cells [45] and other cell types [46], E-cadherin associates with MET and this interaction increased after HGF treatment leading to HGF-MET signaling amplification. Therefore, it is likely that E-cadherin might facilitate the interaction between MET and C3G at the membrane level, as well as HGF/MET signaling. In agreement with this, in a pull-down assay using GST fused to the E-cadherin domain known to interact with C3G, we found interaction with C3G only upon HGF stimulation. In conclusion, C3G is a new key player in HCC tumor growth and progression, correlated with a poor prognosis. Its requirement for a full activation of HGF/MET signaling may offer the possibility to stratify human HCC patients with high C3G expression as putative responders to MET inhibitors.

## 4. Materials and Methods

Detailed procedures can be found in Supplementary materials.

### 4.1. Public Genomic Databases

*C3G* mRNA levels expressed in FPKM (fragments per kilobase of exon model per million reads mapped) was obtained from a TCGA (The Cancer Genome Atlas) cohort of 365 patients, available at The Protein Atlas Platform (https://www.proteinatlas.org/. Patients were divided into four groups depending on their HCC stage following AJCC (American Joint Committee on Cancer) guidelines. A Kaplan–Meier survival curve was performed to compare patients belonging to the above cohort according to high and low *C3G* mRNA expression (median was chosen as cut point).

Tumor and metastasis patient data (HCC liver primary tumor with lung metastasis and their corresponding lung metastasis secondary to HCC liver tumor from 3 and 12 patients, respectively) were provided by the HCMDB (Human Cancer Metastasis Data Base [59]; https://hcmdb.i-sanger.com/) with data collected from NCBI Gene Expression Omnibus and TCGA datasets, expressed in the same units (log2 MAS 5.0 signal) to able the comparison between different experiments and datasets.

### 4.2. Cell Lines and C3G Silencing

Hep3B cells were grown in DMEM, whereas HLE cells in RPMI-1640, both supplemented with 10% fetal bovine serum (FBS) at 37 °C and 5% CO_2_. Mouse *Alb-R26^Met^* HCC cell lines (mHCC1, mHCC13, and mHCC14), established from distinct *Alb-R26^Met^* tumors, previously characterized, were grown in DMEM supplemented with 10% FBS [34,35].

C3G was stably knocked-down by infection with human C3G shRNAs Lentiviral Particles (75,000 infectious units) containing a mixture of different shRNAs (Santa Cruz Biotechnology, sc-29863-V) in the presence of 10 μg/mL polybrene (Santa Cruz Biotechnology, sc-134220) or a control shRNA for non-silenced cells and selected with puromycin (2 µg/mL). A pool of silenced cells was used for the experiments.

To trigger HGF/MET signaling cells were treated with HGF (40 ng/mL; R&D, Minneapolis, MN, USA, 2207-HG-025). The chemical inhibitors used were: SB203580 (5–10 µM; Calbiochem, San Diego, CA, USA, #559389), and SB431542 (10 µM; Sigma, 616464) to inhibit p38α/β MAPK and TGFβ receptor, respectively.

### 4.3. Cell and Tissue Extracts Preparation and Western Blot Analysis

Cells were lysed in an immunoprecipitation (IP) buffer containing 50 mM Tris·HCl (pH 7.5), 150 mM NaCl, 1% NP40, 5 mM EGTA, 5 mM EDTA, 1 mM phenylmethylsulfonyl fluoride, 10 μg/mL aprotinin, 10 μg/mL leupeptin, 1 mM NaVO_3_ and 20 mM NaF to obtain total protein extracts. RIPA buffer was used for tissues. Protein concentration was quantified using Bradford for cell extracts or BCA method for tissue samples. Proteins were separated by electrophoresis using either Anderson [60] or SDS-PAGE gels and transferred to a nitrocellulose membrane as previously described [61]. Then, membranes were probed with primary antibodies listed in CTAT table. β-Actin (Cell Signalling, Leiden, The Netherlands, #3700) or Tubulin (Santa Cruz Biotechnology, sc-2146) were used for normalization. Uncropped Western Blots see Figure S9.

### 4.4. Immunoprecipitation Assay

Total protein extracts (1–2 mg proteins) obtained in IP buffer were incubated with the primary antibody (diluted 1:25 or 1:50) on ice for 3 h. Then, 40 µL of protein A-sepharose (GE healthcare, Chicago, IL, USA, 17-0780-01) (50% *v*/*v*) or protein G-agarose beads (Roche, San Francisco, CA, USA, 11 719 416 001), for rabbit or mouse IgGs, respectively, were added. Samples were rotated for 2–4 h at 4 °C and centrifuged at 14.000 rpm for 1-2 min at 4 °C. Then, beads were washed twice with IP buffer and resuspended in 20 µL of Laemmli buffer for Western-blot analysis.

### 4.5. Pull-Down Assay

SH3b C3G domain, SH3 CrkL domain or C3G-binding E-cadherin domain fused to GST (Glutation S-transferase) or GST alone (as a negative control) were incubated by rotation 1–2 h at 4 °C with Glutathione Sepharose 4B beads (Healthcare, Chicago, IL, USA; 4510) and centrifuged 1 min at 3.000 rpm. The pellet was washed twice with PBS plus PMSF and resuspended at a final concentration of 50% *v*/*v*. Then, lysates (1–2 mg of protein) were incubated with pre-cleared GST fusion proteins (or GST) bound to glutathione beads for 2 h at 4 °C. After centrifugation at 13,000 rpm for 1 min at 4 °C, the pellet was washed with lysis buffer and finally resuspended in 20 µL Laemmli buffer, boiled 5 min and loaded in an 8% SDS-Page for Western-blot analysis.

### 4.6. Proliferation and Apoptosis Analysis by Flow Cytometry

Hep3B cells were maintained in culture either under adherent conditions or in suspension (in a tube under soft shaking to prevent adhesion) for 6h, in the presence or absence of serum. Cells in suspension were directly pelleted by centrifugation. Attached cells were trypsinized, and medium was collected. Then, both medium and cell suspension were centrifuged at 2500 rpm 5 min at 4 °C, fixed with cold ethanol (70%) and washed twice with PBS. Cells resuspended in PBS were incubated with RNase (50 μg/mL) for 30 min at 37 °C and 0.25 μg/mL propidium iodide was added. Then, cell cycle was analyzed by flow cytometry (FACSCan, Becton Dickinson, NJ, USA).

### 4.7. Cell Adhesion Assay

In total, 50,000 cells per well were seeded in 12 multiwell plates with a medium supplemented with 10% FBS and maintained at 37 °C and 5% CO_2_ for 15 or 30 min. Then, the medium was removed, cells were washed twice with PBS and stained with 0.2% crystal violet in 2% ethanol. After washing with distilled water, cells were left to air dry before taking photographs under the microscope. The number of cells per well was quantified using Fiji, ImageJ software, 2012 version, VA, USA.

### 4.8. Wound Healing Assay

Confluent cells were pre-treated for 30 min with Mytomicin C (25 µg/mL, Sigma-Aldrich, M0503) to inhibit cell proliferation. After washing with PBS, a straight scratch was performed and fresh medium without serum was added. Cells were allowed to migrate for 6–24 h at 37 °C and 5% CO_2_. Migration was monitored by phase-contrast microscopy (Eclipse TE300 Nikon, Tokio, Japon, coupled to a digital camera). Photographs were taken to quantify (using TScratch program version 1.0 [62]) the percentage of wound healing closure at different times referred to the value at time 0.

### 4.9. Invasion Assay

Invasion was assayed using transwells (8μm filter, BD, Franklin Lakes, NJ, USA 353097) coated with matrigel (444 μg/cm^2^) (BD Biosciences, Franklin Lakes, NJ, USA, 356234). In total, 50,000 cells were seeded in serum-free medium in the upper chamber. In the lower chamber, 10% FBS medium was added to act as chemoatractant. After 24 h at 37 °C and 5% CO_2_ in a humidified atmosphere, medium and Matrigel from the upper chamber were removed, cells were fixed with 4% paraformaldehyde (PFA), stained with 0.2% p/v crystal violet (Sigma-Aldrich, C-0775) and counted. Microscopy photographs (Eclipse TE300, Nikon) were taken and cells were counted using Fiji, ImageJ software, 2012 version, Dresden, Germany.

### 4.10. Anchorage-Dependent Growth

One-hundred cells were seeded in a 6 cm dish with complete medium and maintained in culture at 37 °C and 5% CO_2_ for 10–15 days. Then, the medium was removed, and cells were washed twice with PBS. Foci were stained with 0.2% crystal violet and washed with distilled water. Microscopy photographs were taken, and the number of foci were assessed using Fiji ImageJ software 2012 version, VA, USA.

### 4.11. Anchorage-Independent Growth

For the anchorage-independent growth, 24-multiwell dishes were pre-coated with 0.5% agar (BD, Franklin Lakes, NJ, USA, 214530) in complete medium. Cells (3 × 10^3^), re-suspended in 0.35% agar diluted in complete medium, were seeded on the top. Fresh medium was added every 3 days, and after 2 weeks, colonies were stained with 0.005% crystal violet and counted using a microscope (Eclipse TE300, Nikon). The number of foci was assessed using ImageJ software.

### 4.12. Induction of EMT with TGFβ

Hep3B cells were serum deprived and treated for 48h with TGFβ (5 ng/mL; Preprotech, London, UK, AF-100-21C) for EMT induction. Thereafter, they were maintained in culture with a chronic treatment with TGFβ (2.5 ng/mL) for phenotype maintenance (stable EMT induced by chronic treatment).

### 4.13. RT-qPCR Analysis

RNA was isolated using RNA isolation kit (Mackerey Nagel, Düren, Germany, MN22740955) following manufacturer instructions, and 1–3 µg of total RNA was reverse-transcribed into cDNA using SuperScript III RT Kit (Invitrogen, Waltham, MA, USA, 18080-040). Real time PCR reactions were performed using specific primers (see Supplementary Materials) and Fast Start Universal SYBR Green Master (Rox) (Roche, San Francisco, CA, USA, 04913850001) to detect DNA by the 7900 Fast Real Time System (Life Technologies, CA, USA, 4329001). GUSB was used as the housekeeping normalizing gene.

### 4.14. Xenograft Assays

Hep3B cells with and without C3G knock-down (10^6^ cells/100 µL) were subcutaneously injected into the flanks of male nude mice (Envigo). Tumor size was monitored twice a week using a caliper and calculated by the formula: (L/2) × (W/2) × π. At the end point, the mice were sacrificed, and tumors were resected and frozen or fixed in 4% PFA for paraffin embedding. Bone marrow and lungs were isolated and processed as indicated in the supplementary information. All animal experiments were carried out in compliance with the European Community Council Directive (2010/63/EU) and following the guidelines for animal research from Complutense University Ethical Committee, approved by Comunidad Madrid (Spain) with reference PROEX028/17; and Ethical Committee from Marseille under an agreement number E1305521 and Project authorization APAFIS #8214-2016121417291352.v5 from French Ministry of Higher Education, Research and Innovation. For the animal study, no randomization was used and no blinding was performed.

### 4.15. Generation of Lung Metastasis in Mice Using the Alb-R26^Met^ HCC Cell Lines

Mouse *Alb-R26^Met^* HCC cell lines (mHCC1, mHCC13, and mHCC14) were subcutaneously injected (5 × 10^6^ cells) into mice flanks (syngeneic transplantation). Tumor growth was followed overtime. In anesthetized mice, tumors were surgically removed before reaching approximately 10 mm diameter, and processed for analyses. After the surgery, mice were regularly followed for about 4 months, then sacrificed to analyze metastases in dissected lungs.

### 4.16. DEN-Induced Liver Tumors

Liver tumors were induced with DEN (Diethylnitrosamine) in C57BL/6 male (*n* = 6 per condition). Male mice at day 15 of age received a single intraperitoneal injection of DEN (10 mg/kg) diluted in saline buffer. At 9 and 12 months (Figure S1B) after DEN injection, mice were euthanized and their livers removed. At those times, all livers had visible tumors. As a control, saline solution was administered. Paraffin-embedded liver sections were prepared from mice 9 months after treatment.

### 4.17. Tissue Samples Staining and Analysis

Paraffin-embedded tumor sections were stained by immunofluorescence. They were incubated with the primary antibody (see CTAT) o/n at 4 °C, followed by the secondary antibody (1 h at RT), either anti-rabbit Alexa Fluor 488 or anti-mouse Alexa Fluor 594 (Invitrogen, A32731 and A32744) and with DAPI. Paraffin-embedded lungs sections were stained with Hematoxylin/Eosin for metastasis analysis. Samples were photographed under a microscope (Eclipse TE300, Nikon) and fluorescence intensity or area of metastasis was calculated using ImageJ software.

### 4.18. Statistical Analysis

Data are represented as the mean ± S.E.M. (*n* ≥ 3). Data was tested for normality with either Shapiro–Wilk or Kolmogorov–Smirnov test. When data showed normal distribution, the parametric unpaired Student’s *t*-test was used for the comparison of two experimental groups and one-way ANOVA or two-way ANOVA for comparing more than two groups with one or two variables, respectively, followed by Tukey, Bonferroni or Dunnet multiple comparison test statistical software recommendations. When data did not show a normal distribution, non-parametric Mann–Whitney test was used for comparison of two experimental groups and Kruskal–Wallis test was used for comparing more than two experimental groups, followed by multiple comparison controlling the False Discovery Rate (FDR) using the Benjamini, Krieger and Yekutieli test. Survival analysis was performed using Log-Rank Mantel–Cox test. Differences were considered significant when *p* value was ≤0.05. GraphPad Prims version 6.01 (GraphPad, San Diego, CA, USA) was used for the analysis.

## 5. Conclusions

**Figure 7:**
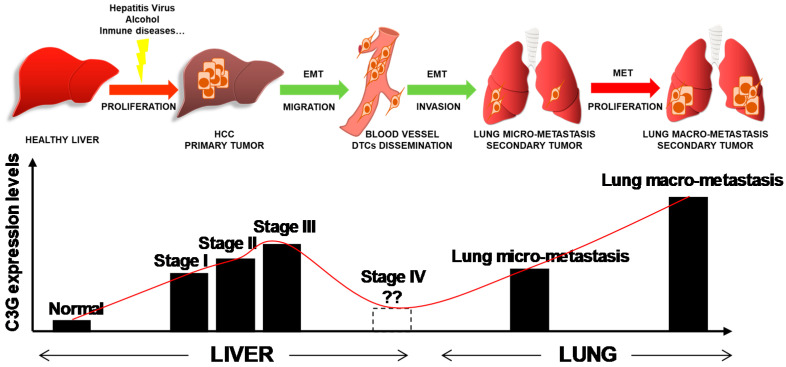
C3G expression from healthy adult liver to HCC tumor growth and progression. Schematic representation of the potential changes in the pattern of C3G expression levels during HCC progression. The progressive increase in C3G would allow HCC primary tumor growth, while a decrease would be associated to EMT and lung metastases generation. Finally, C3G might increase in parallel with lung metastasis growth. Red arrows represent increase and green arrows decrease in C3G levels.

In this work, we uncovered a novel key function for C3G in HCC. We found that C3G expression is gradually upregulated in HCC patients during disease progression (Figure 7). Moreover, these high C3G levels correlate with a lower survival rate in HCC patients. C3G expression is also increased in mouse HCC models and in human and mouse HCC cell lines, and its down-regulation reduces the size of primary HCC tumors in xenograft models by increasing apoptosis and reducing proliferation. The reduction in C3G levels also confers the acquisition of a mesenchymal phenotype with enhanced migratory and metastatic properties to HCC cells, which might favor the generation of lung metastasis (Figure 7). This suggests that C3G expression during HCC is fine tuned in order to facilitate HCC progression. Furthermore, C3G is necessary for the full activation of HGF/MET signaling in HCC cells.

Our results strongly suggest that C3G could be used as a new prognosis biomarker for HCC patients and for stratifying HCC patients for targeted therapy. Hence, patients with higher levels of C3G could be more dependent on MET signaling to sustain tumorigenic properties, and thus putatively better responders to MET inhibitors. Therefore, C3G expression levels could allow classification of HCC patients for MET inhibition-based therapy.

## Supplementary Materials

The following are available online at https://www.mdpi.com/2072-6694/12/8/2282/s1, Figure S1: C3G expression is increased in HCC, Figure S2: Effect of C3G silencing on in vitro tumorigenic ability, invasive capacity and apoptosis in HCC cells, Figure S3: Effect of C3G down-regulation on EMT markers in HCC cells, Figure S3: C3G down-regulation increases apoptosis in human HCC cells, Figure S4: C3G promotes in vivo dissemination of DTCs (Disseminated Tumor Cells) in Bone Marrow, Figure S5: C3G expression increases in lung metastases as compared with primary tumors, Figure S6: C3G interacts with P-MET and Gab1 independently of CrkL, Figure S7: MET is overexpressed in a panel of human hepatocarcinoma cell lines, and Figure S8: C3G knock-down increases p38 MAPK activation, which contributes to C3G knock-down induced migration in Hep3B cells, Figure S9: Uncropped western blots, Supplementary material and methods: Antibodies used for western-blot analysis, primers used for RT-qPCR analysis, β-catenin, apoptotic nuclei and Cleaved Caspase 3 analysis by immunofluorescence, isolation of cells disseminated to bone marrow in xenograft assays, tumor and lung paraffin embedding, public genomic databases, References [63] are cited in the supplementary materials.

## Ackowledgements

The authors thank Cristina Baquero and Nerea Palao for their help to carry out some experiments.
