# Supplementary material for: C3G Is Upregulated in Hepatocarcinoma, Contributing to Tumor Growth and Progression and to HGF/MET Pathway Activation
Source: Cancers (Basel). 2020 Aug 14;12(8):2282. doi: 10.3390/cancers12082282 (PMC7463771; doi:10.3390/cancers12082282)
Supplement: Supplementary file 1 [file cancers-12-02282-s001.pdf]

cancers MDPI

Supplementary Materials: C3G Is Upregulated in Hepatocarcinoma, Contributing to Tumor Growth and Progression and to HGF/MET Pathway Activation

Celia Sequera, Paloma Bragado, Sara Manzano, Maria Arechederra, Sylvie Richelme, Alvaro Gutiérrez-Uzquiza, Aránzazu Sánchez, Flavio Maina, Carmen Guerrero and Almudena Porras

Supplementary Materials and Methods

Antibodies Used for Western-blot an Alysis

Primary antibodies were purchased in Cell Sigaling Technology for Met (8198), P-Met Tyr1234/1235 (3126), P-Abl Tyr412 (2865), Gab1 (3232), P-Gab1 Tyr627 (3231), P-Akt Ser473 (9271), ERKs (9102), P-ERKsThr202/Tyr204 (9101), P-p38 MAPK Thr180/Tyr182 (9211), β-Actin (3700), β-Tubulin (2146), Cleaved Caspase 3 Asp 175 (9661); in Invitrogen for Occludin (71-1500); in Santa Cruz Biotechnologies for: C3G H300 (sc-15359), p38α C-20 (sc-535), CrkL C-20 (sc-319); in BD Biosciences for: E-Cadherin (610181), N-Cadherin (610920), Vimentin (550513); and a custom polyclonal antibody for C3G was generated by Genosphere Biotechnologies (used for blots from Figure 5E); Secondary anti-rabbit (Cell Signaling, 7074, Leiden, The Netherlands) or anti-mouse antibody (Cell Signalling, 7076) conjugated with HRP (Horse Radish Peroxidase).

Primers Used for RT-qPCR Analysis

-Twist1: 5′-CAAAGAAACAGGGCGTGGGG-3′
3′-CAGAGGTGTGAGGATGGTGCC-5′
-Zeb2: 5′-AATGCACAGAGTGTGGCAAGGC-3′
3′- ATCTGGCGTTCCAGGGACTCAT-5′
-C3G: 5′- GGTGCAGAACGATCCTCGAA-3′
3′- AGACCAGCGAATGAGGTTGG-5′
-GUSB: 5′-AAAATGGAGTGCGTGTTGGGTCG-3′
3′-CCACAGTCCGTCCAGCGCCTT-5′

β-Catenin, Apoptotic Nuclei and Cleaved Caspase 3 Analysis by Immunofluorescence

For β-catenin analysis, Hep3B cells were seeded in circular glass coverslips pre-coated with gelatin 2% (Sigma, G9391, San Jose, CA, USA), grown in culture medium supplemented with 10% FBS and serum starved for 24 h. After that, cells were washed with PBS twice, fixed with 4% paraformaldehyde (PFA) (Sigma 158127) in PBS for 20 min at RT.

To detect cleaved-caspase 3 (CC3) and apoptotic nuclei after DAPI staining, Hep3B cells were maintained in culture, either under adherent conditions or in suspension (in a tube under soft shaking to prevent adhesion) for 6h, in the presence or absence of serum. Cells in suspension were directly pelleted by centrifugation, while attached cells were trypsinized and pelleted. Then, cells were fixed with 4% PFA for 20 min at 4 °C and seeded overnight in circular glass coverslips pre-coated with gelatin 2%.

After fixation, in all cases, cells were washed with PBS and permeabilized by incubation for 20 min at RT with 0.1% Triton TX-100 (Sigma X-100) in PBS containing 0.1% BSA (Panreac A1391). Then, cells were incubated with blocking solution (5% BSA in PBS) for 1h at RT. Next, cells were incubated with β-catenin antibody (BD Bioscience, Franklin Lakes, NJ, USA, 610154) at a dilution of 1/50 in 1% BSA PBS or Cleaved caspase 3 (CC3) antibody (Cell signaling #9664S) at a dilution of 1/100 o/n at 4 °C. Then, cells were washed with PBS and coverslips were incubated with anti-mouse FITC

secondary antibody diluted 1/200 (Sigma F0257) for β-catenin and Alexa 488 goat anti-rabbit (Invitrogen, Waltham, MA, USA, A32731) diluted 1/500 for CC3 and DAPI (dilution 1/1000, Panreac A4099) in 0.1% BSA-PBS. After washing with PBS, cells were mounted with mounting medium (Ibidi 50001) and images were captured using an immunofluorescence microscope (Nikon Eclipse TE300) coupled to a camera.

Isolation of cells Disseminated to Bone Marrow in Xenograft Assays

Bone marrow (BM) from tibiae and femur was flushed with 5 mL PBS using a 26G needle and centrifuged at 1200 rpm RT. BM suspension was slowly deposed on the surface of Percoll (5 mL) and centrifuged without brake at 1500 rpm for 30 min. Cells were recovered, with 10 mL PBS and centrifuged at 1500 rpm 5 min at RT. BM flushing was fixed with 4% PFA in PBS for 20 min at 4 °C and centrifuged at 1200 rpm for 5 min. Pre-treated slides were mounted with paper pad and the cuvette in the metal holder, then 100 µL of the cell suspension (10^6 cells/mL in 1% BSA-PBS) was loaded in each cuvette. Samples were cyto-spun at 500 rpm for 3 min, and cytospin slides were carefully extracted and were allowed to dry for 5 min and stored in PBS at 4 °C.

Tumor and Lung Paraffin-Embedding

Tissue samples fixed in 4% PFA o/n were washed in cold PBS (2 × 5min) and dehydrated by incubation in a rocker at 4 °C: 3 × 20min in cold 30% EtOH; 3 × 20min in cold 50% EtOH; 3 × 20min in cold 70% EtOH; 95% EtOH o/n; 3 × 20min in 100% EtOH at RT; and 3 × 15min in xylene at RT. Then, samples were infiltrated with paraffin wax by successive steps at 62 °C as follows: 30min in paraffin: xylene (1:1); 3 × 1h in paraffin; finally, samples were placed in embedding moulds with fresh paraffin and topping with a plastic cassette. Block sections were performed with the microtome (5–7 µm) and mounted into APES pre- coated slides.

Public Genomic Databases

Data for the analysis of Rapgef1 mRNA levels in primary human adult hepatocytes (hHep-A), human adult liver (hLiver-A), human fetal liver (hLiver-F), human hepatic progenitor cells (hHPCs) and human HCC cell lines were obtained from NCBI GEO datasets accession number GSE112330 and GSE83518 and analyzed by GREIN platform (GEO RNA-seq Experiments Interactive Navigator (http://www.ilincs.org/apps/grein/) [63]

For the analysis of Rapgef1 mRNA expression in normal and DEN-induced mouse liver cancer samples, data were provided by NCBI GEO dataset, accession number GSE93392.

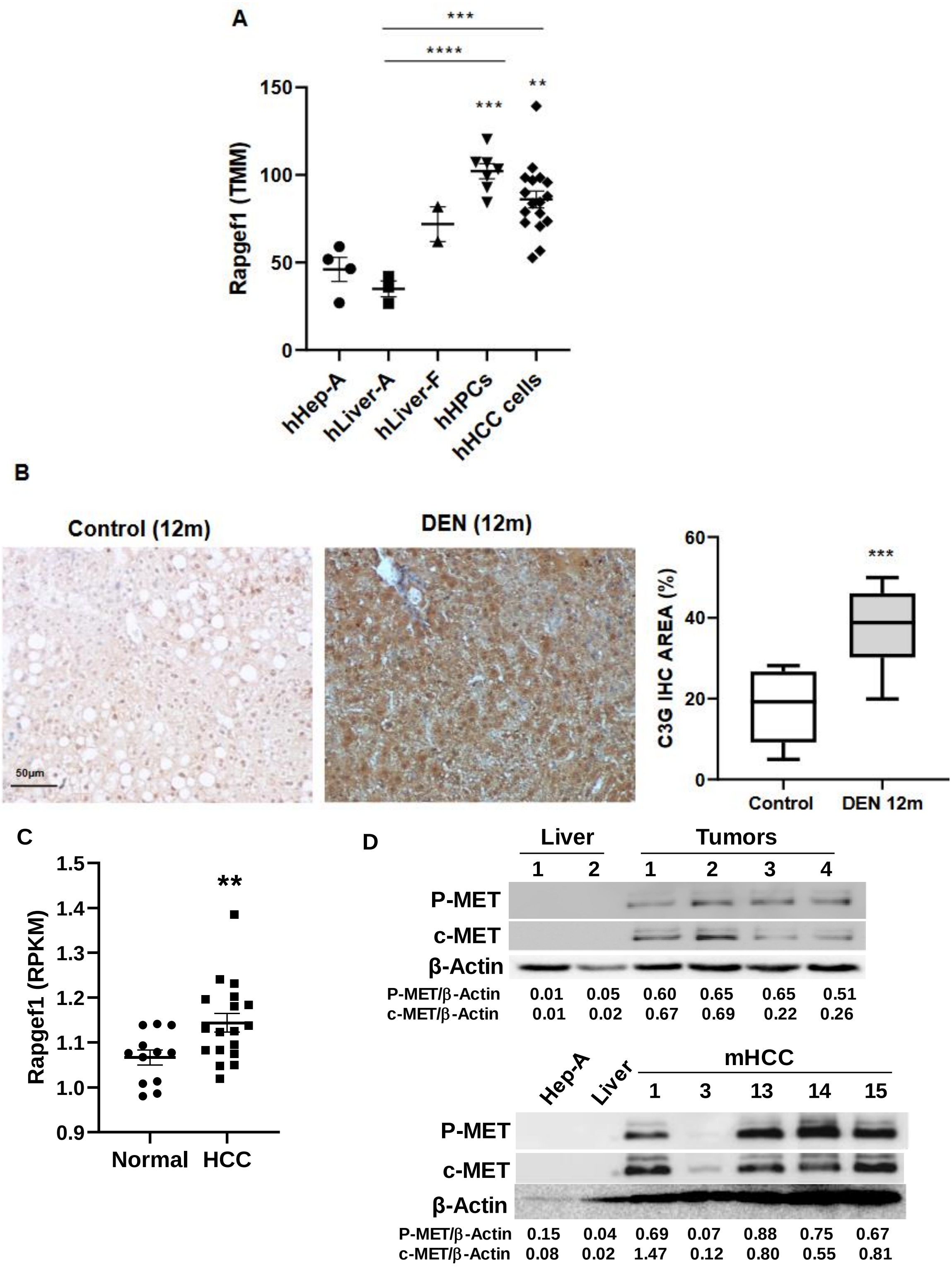

Figure S1. C3G expression is increased in HCC. (**A**) Scatter plot of Rapgef1 mRNA levels expressed and normalized as TMM (Trimmed Mean of M-values) of primary human adult hepatocytes (hHep-A), human adult liver (hLiver-A), human fetal liver (hLiver-F), human hepatic progenitor cells (hHPCs) and human HCC cell lines (Hep3B, HLE, HLF, HepG2, Huh1, Hep293TT, Huh6 and HepU2). ** $p \leq 0.01$, *** $p \leq 0.001$, **** $p \leq 0.0001$, One-way ANOVA followed by multiple comparison, versus Hep-A or as indicated. (**B**) Representative images of C3G levels analyzed by immunochemistry in liver tumor sections from control (no treatment) and DEN-treated mice at 12 months after treatment; right panel, box plot showing the mean value ± S.E.M. of C3G positive areas, analyzed by ImageJ. (**C**) Scatter plot of Rapgef1 mRNA expression in normal and DEN-induced mouse liver cancer samples

expressed in RPKM. ** $p \leq 0.01$, *** $p \leq 0.001$, Student-t test, versus normal samples. (**D**) Western-blot analysis of total Met and P-Met normalized with β-actin in: (upper panel) control healthy liver and *Alb-R26^{Met}* tumors (1, 2, 3, 4) and (lower panel) adult hepatocytes (Hep-A), control healthy liver and HCC cells established from *Alb-R26^{Met}* HCC tumors (mHCC1, 3, 13, 14, and 15).

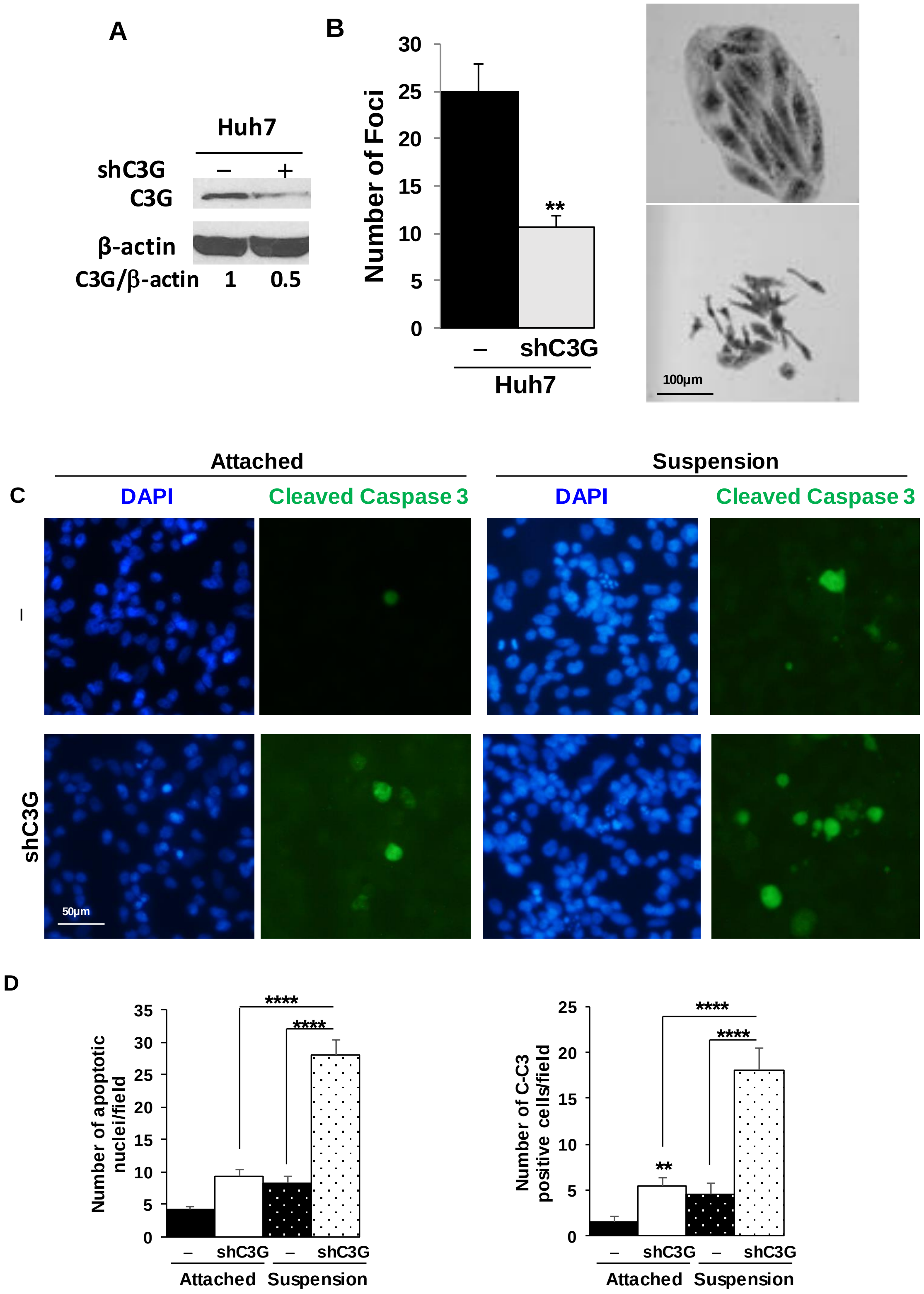

Figure S2. Effect of C3G silencing on in vitro tumorigenic ability, invasivecapacity and apoptosis in HCC cells. (**A**) and (**B**). Huh7 (non-silenced (–) and C3G knock- down (shC3G)) were maintained in complete medium. (**A**) Western-blot analysis of C3G expression normalized with β-actin, $n = 2$. (**B**) Anchorage-dependent growth assay. Left panel, histogram showing the mean ± S.E.M. of the number

of foci. Right panel, representative microscope photographs of individual foci (n = 3). (**C**) Representative microscopy images of the immunoflorescence staining of nuclei with DAPI and active caspase 3 in Hep3B cells maintained attached or in suspension in serum-free medium. **(D)** Histograms show the number of apoptotic nuclei per field (left panel) and the number of active caspase 3 (C-C3) positive cells per field (n = 5). ** $p \leq 0.01$, *** $p \leq 0.001$, **** $p \leq 0.0001$ versus attached non-silenced cells (−) or as indicated analyzed by One-way ANOVA, followed by multiple comparison, $n \geq 3$.

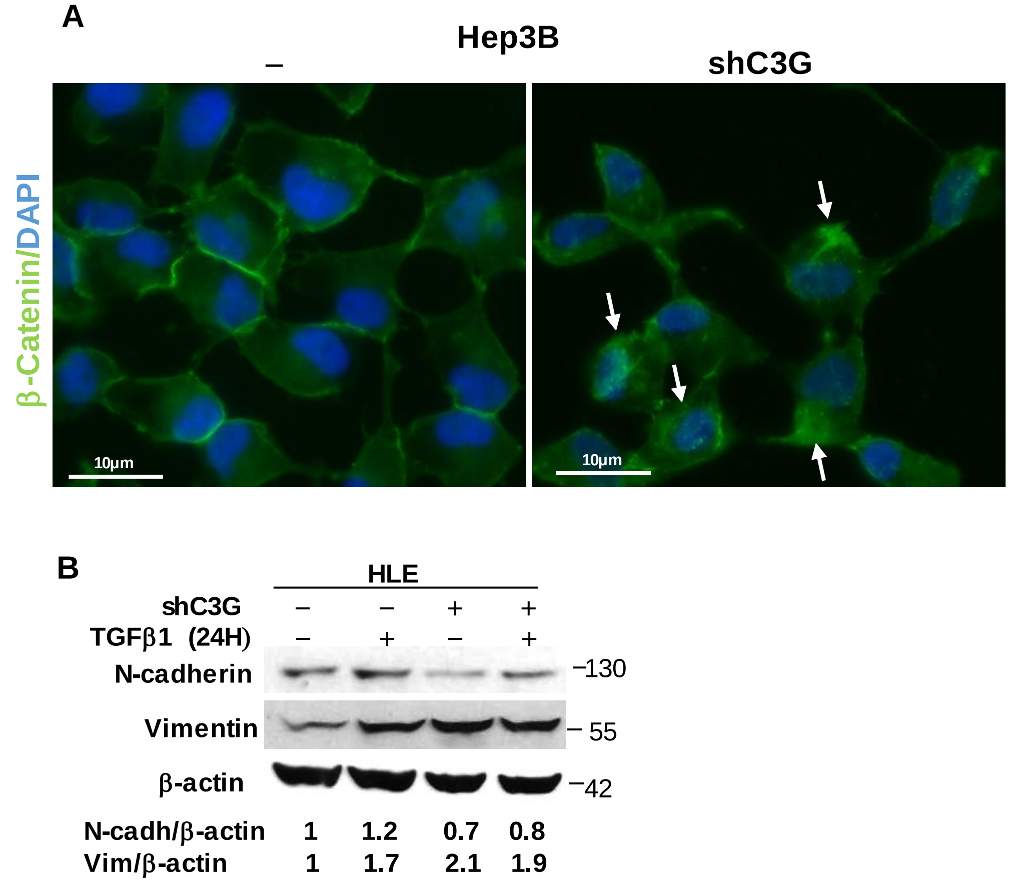

Figure S3. Effect of C3G down-regulation on EMT markers in human HCC cells. (**A**) Representative immunofluorescence microscopy images of β-catenin (green) in Hep3B cells with (shC3G) or without (−) C3G knock-down, under serum-starved conditions. Nuclei were stained with DAPI (blue). White arrows point at internalized β-catenin. (**B**) Western-blot analysis of mesenchymal markers, N-cadherin and Vimentin, normalized with β-actin, in HLE (non-silenced (−) and C3G knock-down (shC3G)) with (+) or without (−) an acute (24 h) treatment with TGF-β1, n = 3.

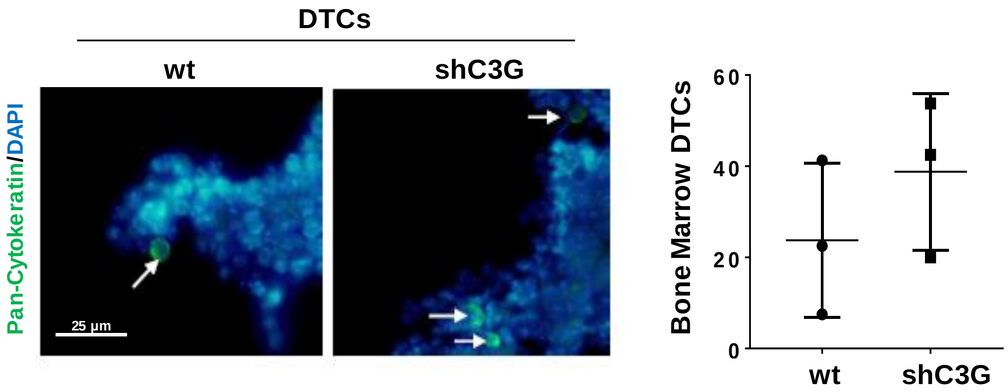

Figure S4. C3G promotes in vivo dissemination of DTCs (Disseminated Tumor Cells) in Bone Marrow. Cytospin of bone marrow obtained from nude mice injected with Hep3B (non-silenced (−) and C3G knock-down (shC3G)) cells. Immunofluorescence staining with Pan-cytokeratin (green) and nuclear DAPI (blue). Left panel, representative images, white arrows indicate positive cells. Right panel, scatter plot of the mean ± S.E.M of Bone Marrow DTCs, analyzed as positive pan-cytokerakin cells (n = 3).

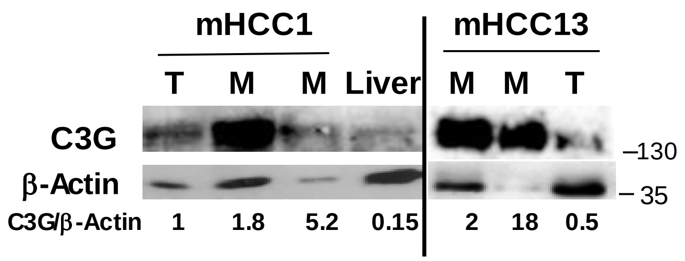

Figure S5. C3G expression increases in lung metastases as compared with primary tumors. Mouse Alb-R26Met HCC cell lines were injected subcutaneously into mice flanks to generate tumors and lung metastasis. Western-blot analysis of C3G levels in control liver tissue, tumors and lung metastasis generated by mHCC1 and mHCC13 cells normalized with β-actin (n = 4).

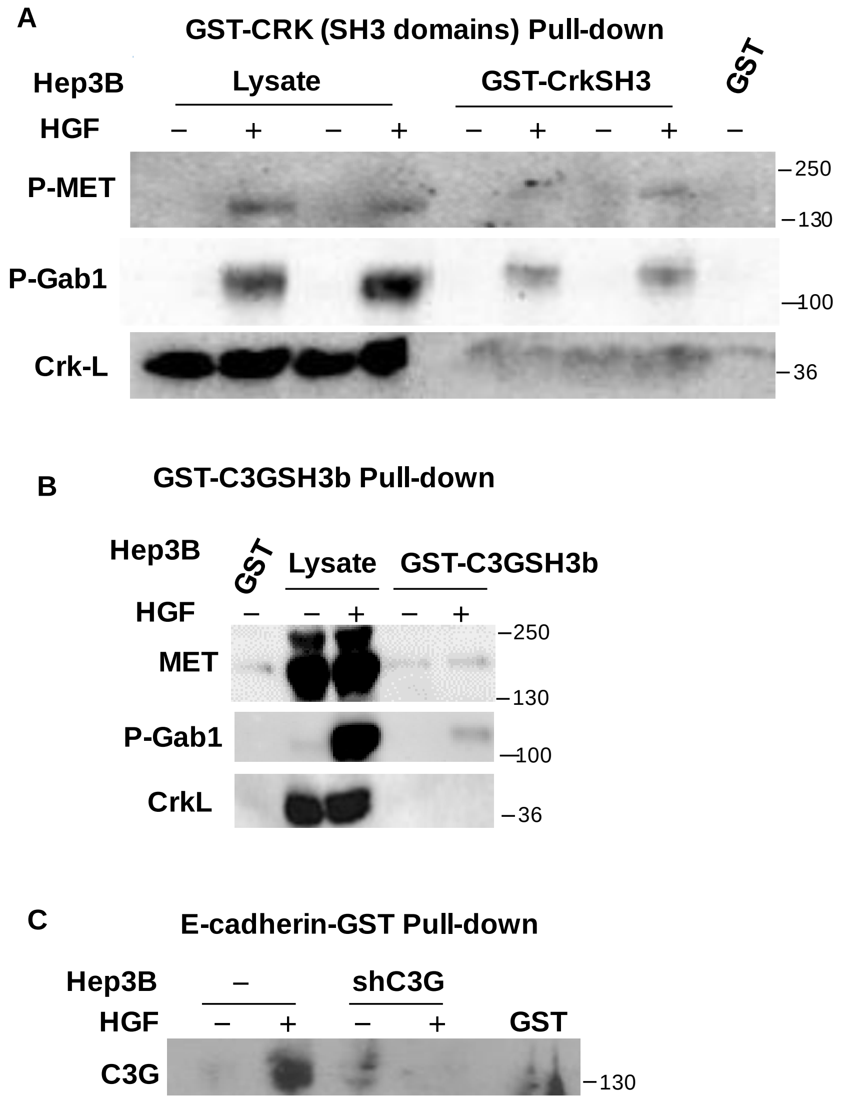

Figure S6. C3G interacts with P-MET and Gab1 independently of CrkL. Hep3B cells were serum starved for 24 h and stimulated with HGF for 5min or maintained untreated. (**A**) Western-blot analysis of P-MET, P-Gab1 and CrkL in total lysates and in samples from pull-down assays performed with

GST fused to Crk SH3 domains (GST-CrkSH3), *n* = 3. (**B**) Western-blot analysis of c-MET, P-Gab1 and CrkL in lysates and samples from pull down assays performed with GST fused to C3G SH3-binding domain (GST-C3GSH3b), *n* = 3. (**C**) Western-blot analysis of C3G in lysates and samples from pull down assays from non-silenced (−) and C3G knock-down (shC3G) Hep3B cells performed with GST fused to E-cadherin domain known to bind C3G (GST-E-CadC3Gb), *n* = 2 (**A**–**C**) As a negative control, lysates were incubated with GST-beads.

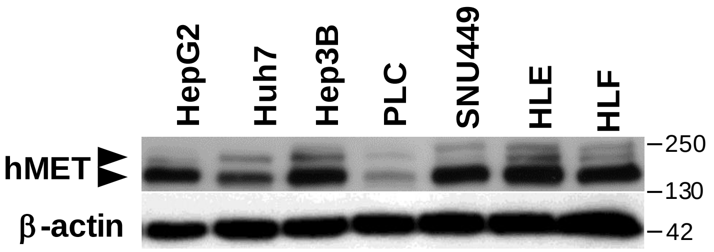

Figure S7. c-MET is overexpressed in a panel of human hepatocarcinoma cell lines. Western-blot analysis of human c-MET protein levels in several human HCCcell lines, normalized with β-actin.

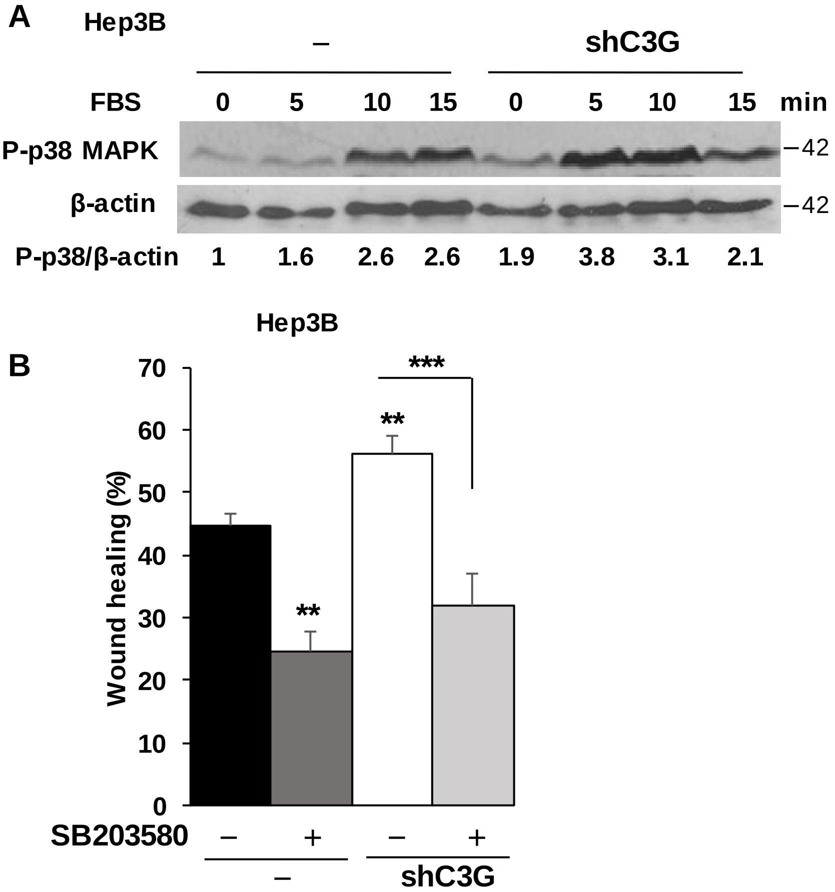

Figure S8. C3G knock-down increases p38 MAPK activation, which contributes to C3G knock-down induced migration in Hep3B cells. Hep3B (non- silenced and C3G knock-down (shC3G)) cells were used. (**A**) Cells were serum-starved and stimulated with 10% FBS for 5-15min. Activation of p38 MAPK was determined by western-blot analysis of P-p38 MAPK levels and normalized with β-actin ($n = 3$). (**B**) Wound healing assay. Cells were maintained in the absence of serum, untreated (−) or treated with the p38 MAPK inhibitor, SB203580 (5 µM) and were allowed to migrate after doing the wound. Histogram represents the mean ± S.E.M. of wound closure percentage (%). ** $p \leq 0.01$, *** $p \leq 0.001$, versus non-silenced cells (−), Two-way ANOVA, followed by multiple comparison, $n \geq 3$.

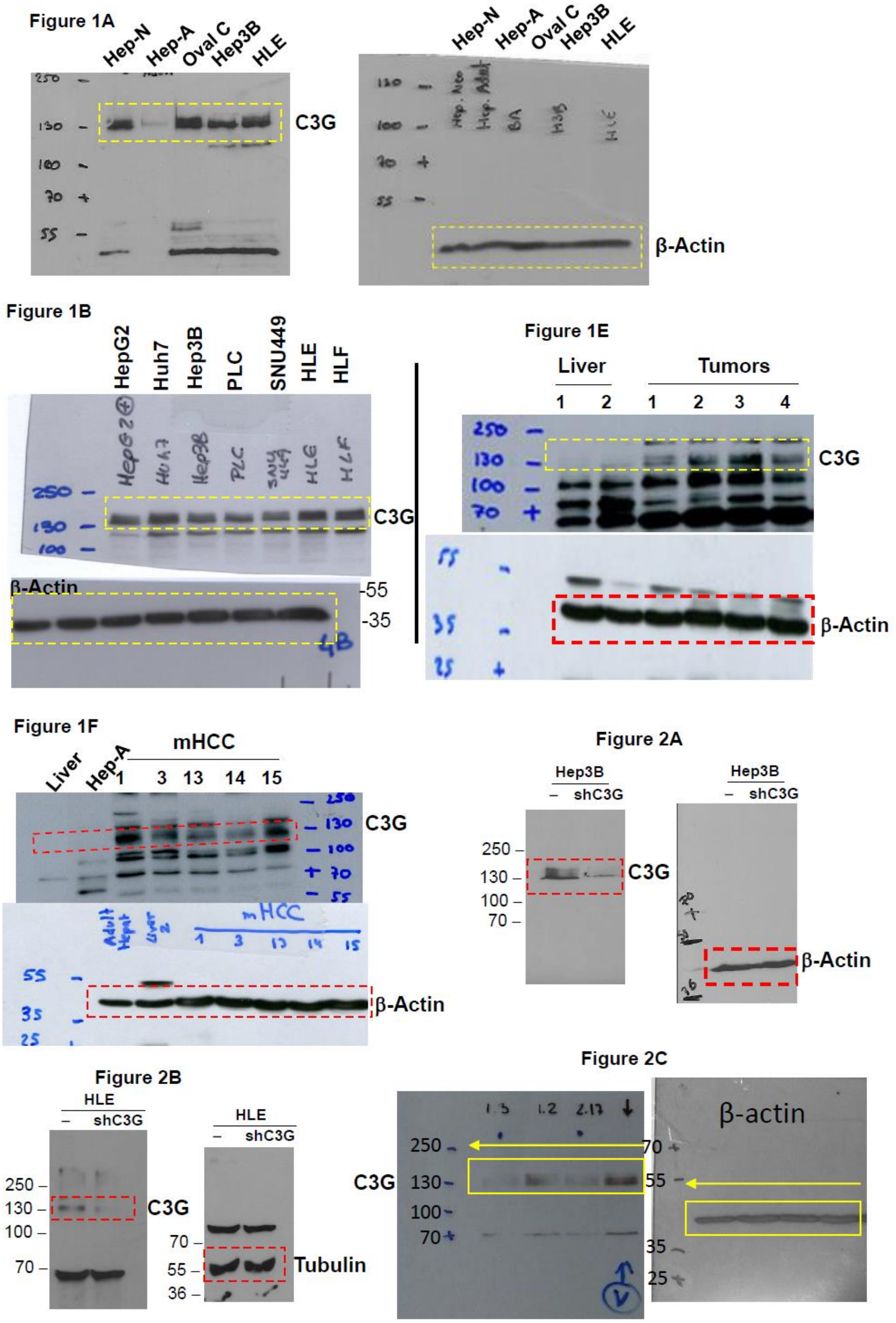

Figure S9. *Cont.*

Figure 3C

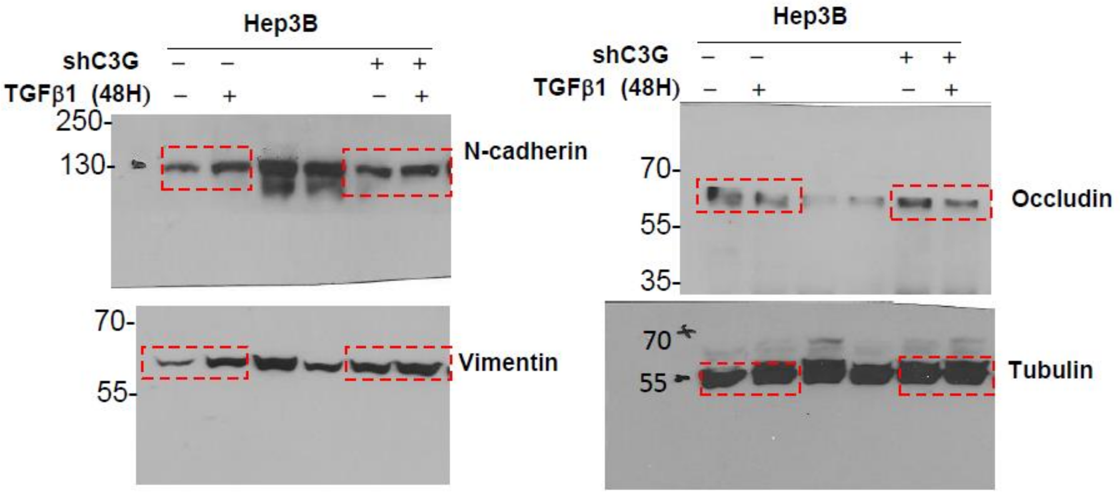

Figure 5E

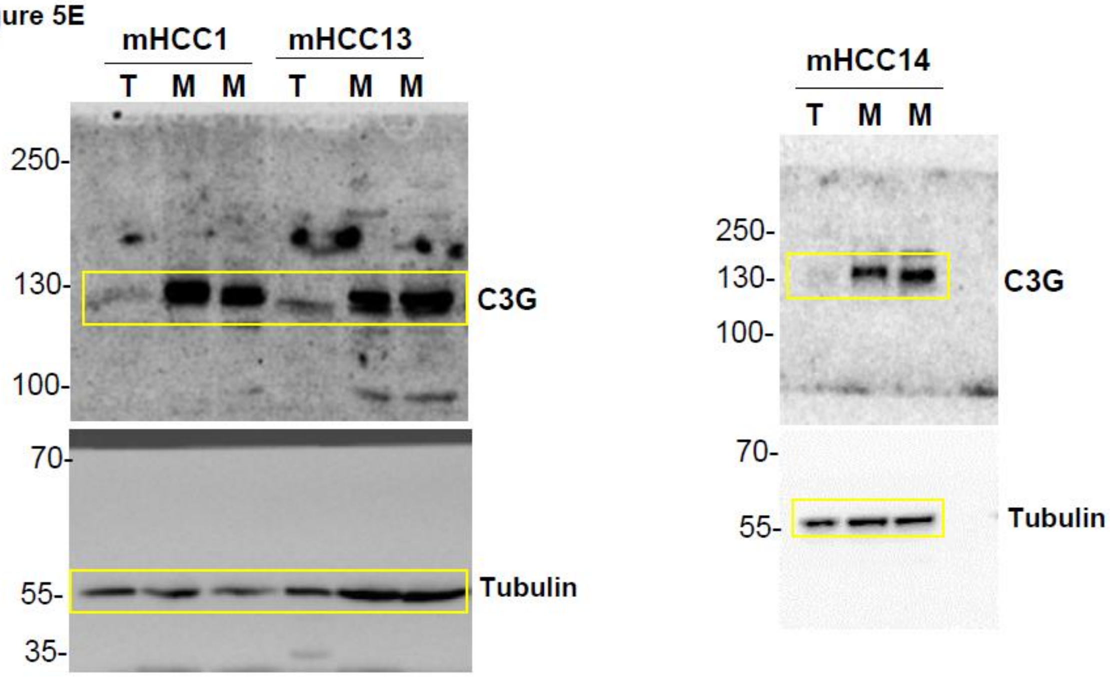

Figure S9. *Cont.*

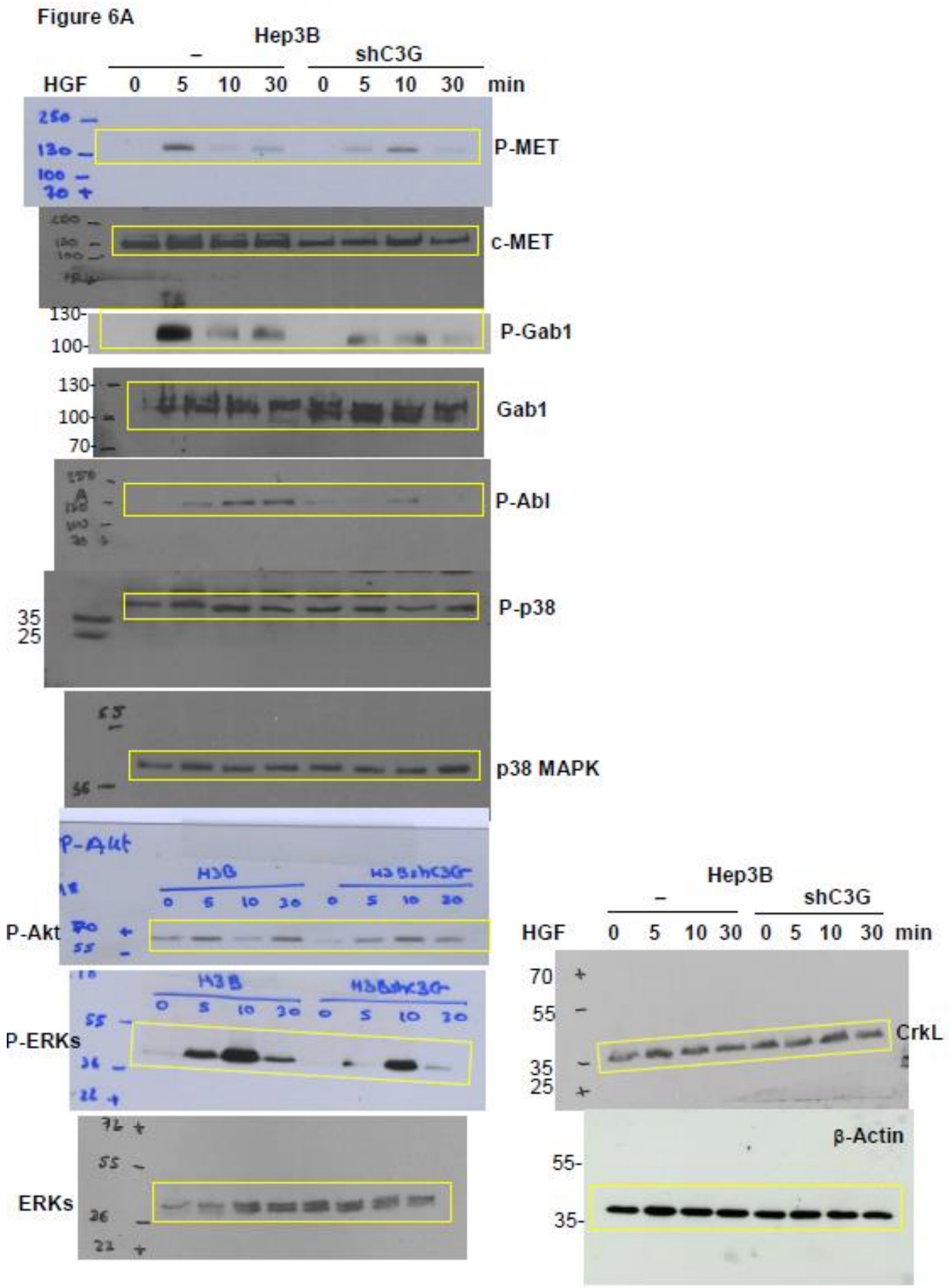

Figure S9. *Cont.*

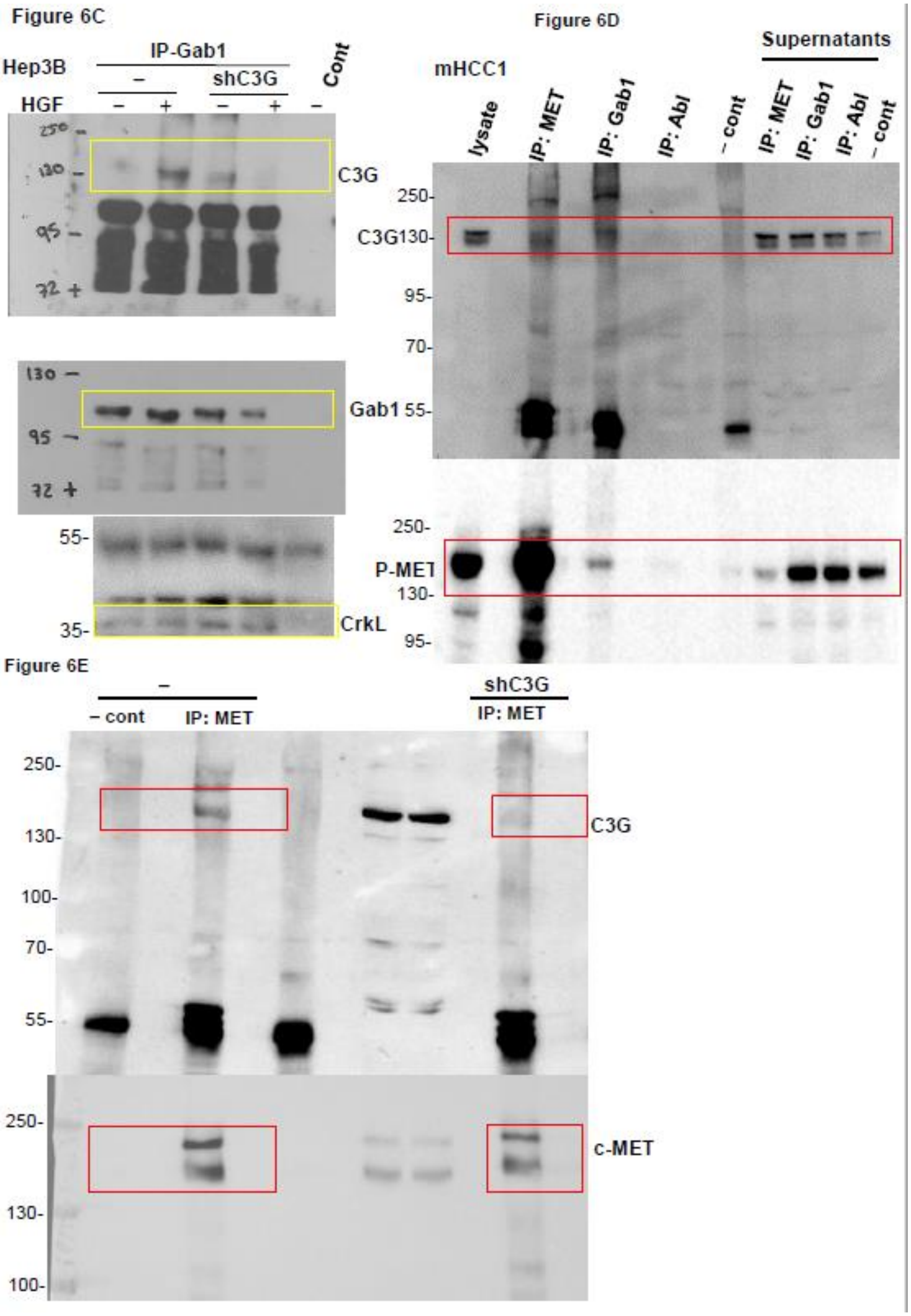

Figure S9. *Cont.*

Figure S1A

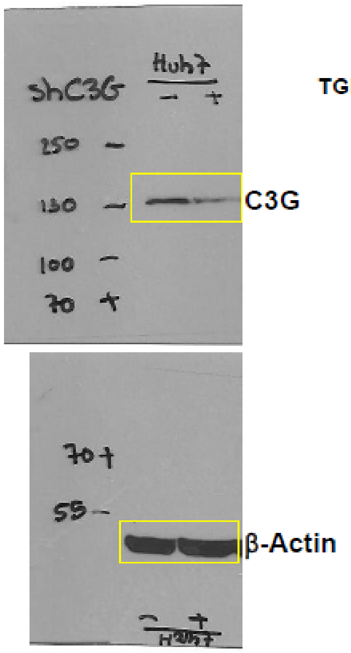

Figure S1D

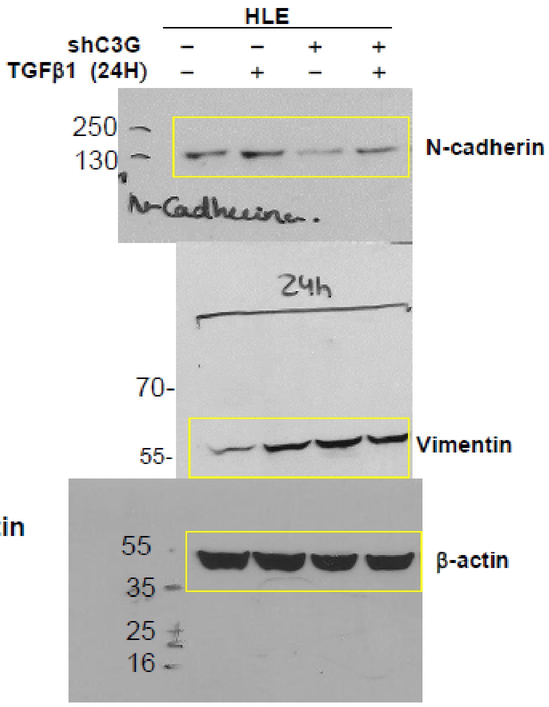

Figure S3

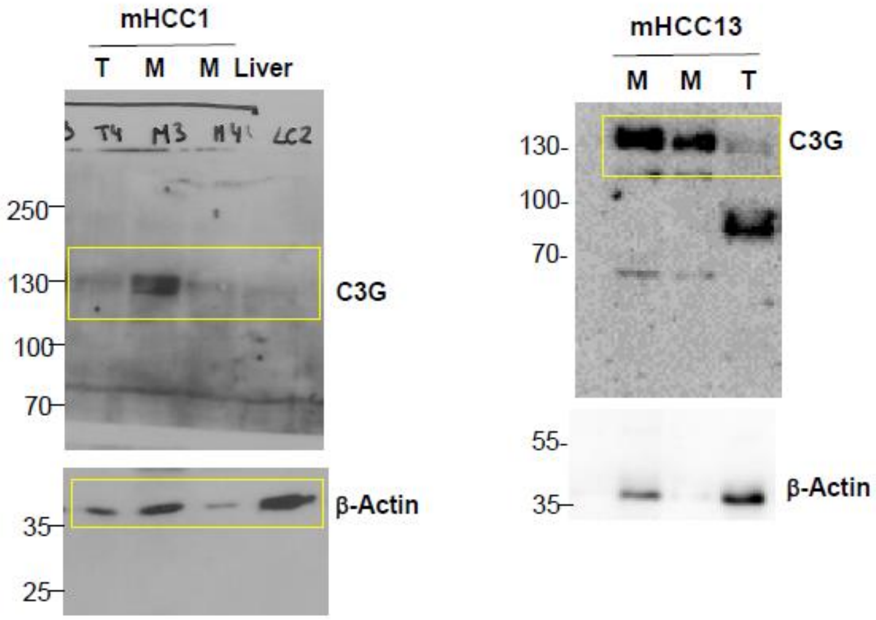

Figure S9. *Cont.*

Figure S4A

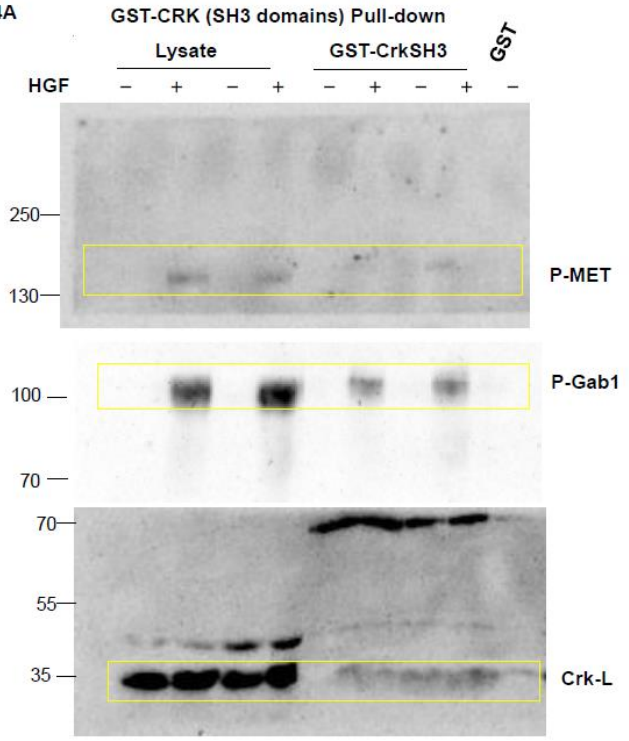

Figure S4B

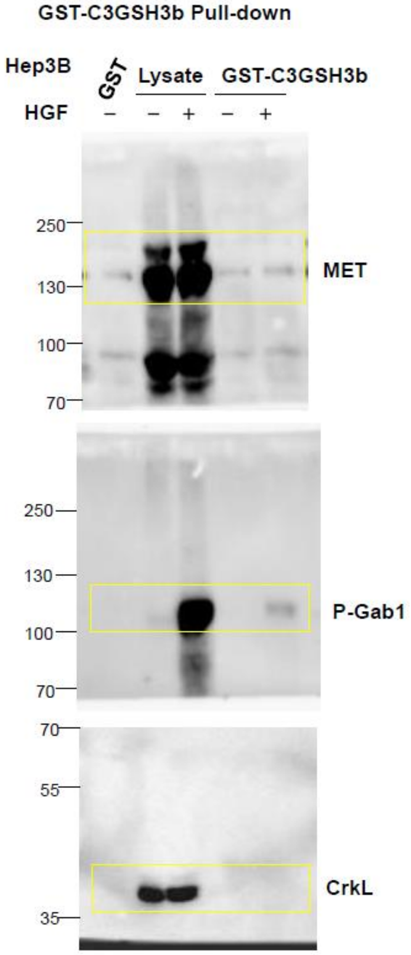

Figure S4C

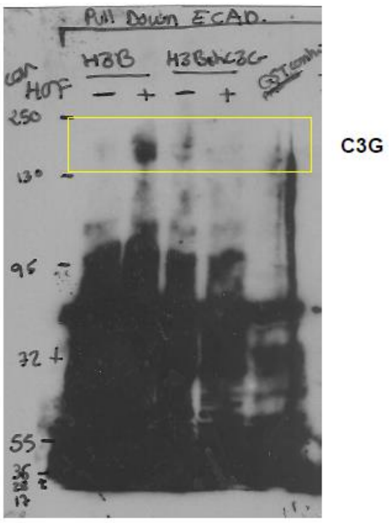

Figure S9. *Cont.*

Figure S5

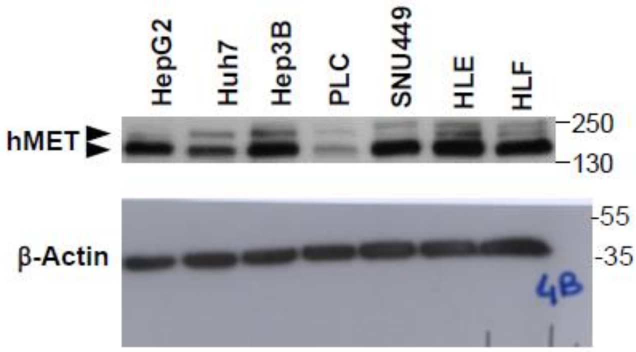

Figure S6A

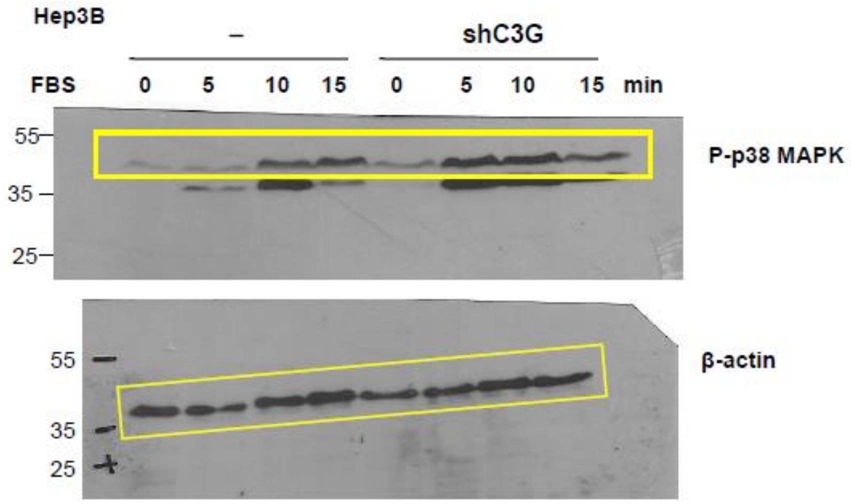

Figure S9. Uncropped Western Blots.

© 2020 by the authors. Licensee MDPI, Basel, Switzerland. This article is an open access article distributed under the terms and conditions of the Creative Commons Attribution (CC BY) license (http://creativecommons.org/licenses/by/4.0/).
